# 

**Published:** 1876-12

**Authors:** 


### CONTENTS VOL. XXX.

### A.

A well marked case of Rubber Poison cured by substituting a Celluloid Plate . . . 25
A Case in Practice . . . . 32
Alveolar Abscess . . . . . 61
A day not very far from the Alps, Switzerland . 91
American Dental Association . . . 97
Amalgam Fillings . . . . . 119
A Severe Case . . . . . 143
Abrasion of the Teeth with Sensitiveness . 145
Address . . . . . . 149
A Good Name . . . . . 184
Annual Address . . . . . 193
Artificial Separation of the Teeth as a Means of Prevention of Decay . . . . 207
Address . . . . . . . 217
Avoidance of Phosphorus Poisoning . . 224
American Dental Association . . 323, 236
American Academy of Dental Science . . 323

Artificial Separations, versus Extraction of six year Molars . . . . . . 342
A Rag File . . . . . . 367
A Lady D. D. S. . . . . . 409
A new Saliva Extractor . . . . 456
A New Forceps . . . . 535

**B.**

Biographical . . . . . 138
Bad Taste . . . . . . 447

**C.**

Constitutional Relations of the Teeth . . 13
Commencement . . . . 232, 83
Conn. Valley Dental Association . . 449, 226
California State Dental Association . . 275
Correspodence . . . . . 277
Celluloid . . . . . . 318
Causes of Decay of Teeth . . . . 339
Celsus . . . . . . 454

**D.**

Dental Manipulation . . . . 26
Diseases of the Gum and Treatment . . 52
Dental Hygiene . . . . . 170
Died . . . . . . . 233
Dental Announcement . . . . 278
Dental Caries and the Remedy . . . 292
Discussions of the Mississippi Valley Dental Society 345, 307
Dental Society of the State of New York . . 320
Dental Histology . . . . . 392
Dental Therapeutics . . . . . 426
Dental Education . . . . . 485
Dental Legislation and its Necessities . . 501
Dental Pathology . . . . . 515
Dental Education. No. 1 . . . . 522
Diamond Disks . . . . . 535

E.

Evils from the use of Wheaten Flour . . . 45
Extracts from the Transactions of the Am. Academy of Dental Surgery . . . 123
Essential Elements of Success in Dental Practice 385
Educational—Students . . . . 408
Extract from the Proceedings of the Miss. State Dental Society . . . . . 444

F.

Filling Teeth with Tin . . . . 9
Fluid extract of Gelseminum as a Cure of Neuralgia of Fifth pair of Nerves . . . 144
Fragmentary Clippings . . . . 287
Filling Temporary Teeth . . . . 343
Fracture of the Facial Bones . . . 400
"Fragmentary Clippings" . . . . 451
Fiat-Justitia . . . . . . 452

H.

Hydroadipsia and the Water Supply of Living Bodies Clinically Illustrated . . . 252

I.

Instruments used in the Extraction of Teeth . 16
In Memoriam . . . . 335, 104
Iowa State Dental Society . . 227, 148
Illinois Dental Society . . . . 185
Indiana State Dental Society . . . 276

K.

Kentucky State Dental Society . . 324, 226

L.

Legislation for Regulating Dental Practice . 101
Local and Constitutional Treatment of Diseases . 113
Lawrenceburg, Ind. , . . . . 190
Lamps . . . . . , 237
Lister's Treatment of Wounds by the Antiseptic Method . . . . . . 264
Law Pertaining to Dentistry . . . 315
Local Anæsthesia . . . . . 533

### M.

Michigan University Dental Department . 147
Mad River Dental Society . . . 448, 148
Minutes Miss. Valley Dental Society . . 175
Miss. Valley Dental Society . . . 182
Micro-Photographs . . . . . 232
Michigan University Medical College . . 272
Micro-Photographs in Histology, Normal and Pathological . . . . . . 497
Mechanical Dentistry . . . . . 506

### N.

Northern Ohio Dental Society . . . 277
New Jersey State Dental Society . . . 322

### O.

Oregon State Dental Society . . . 35
Obituary . . . . . . 103
Ohio Dental College Association Commencement 105
Ohio College Faculty Changes . . . 193
Oral Electricity . . . . . 295
Ohio State Dental Society . . . . 357
Ohio State Dental Society . . . . 366
Obituary . . . . . . 368
Observations upon the Natives of the Hawaiian Isles . . . . . . 369
Obituary . . . . . . 410
Operative Dentistry . . . . . 473 418
Ohio State Dental Society . . . . 496
Ohio State Dental Society . . . . 500
Observation on Hare-lip and Cleft Palate . 530
On the Salicylate of Sodium . . . 532

### P.

Peroxide of Hydrogen as a Deodorizer . . 46
Proceedings of Central Ohio Dental Society . 100
Prof. Taft's criticisms . . . . 166
Proceedings of Mich. State Dental Society . 221
Postponement . . . . . 279
Personal . . . . . . 324 280

Physiology . . . . . . 325
Prevention and Treatment of Approximate Decay 375

Q.

Questions on the Structure and Development of Teeth . . . - . . 60
Query . . . . . . 117
Quackery . . . . . 404

R.

Replacing Extracted Teeth . . . 50
Reflex Nervous Disorders from Dental Irritation . 132 70
Report on Dental Physiology . . . 214
Report on Therapeutics . . . . 333
Replanting a Tooth . . . . 364

S.

Southern Dental Association . . . 183
Salicylic Acid . . . . . 191
Stammering a Hereditary Defect . . . 362
Seventh Annual Meeting of Northern Ohio Dental Association . . . 434
Special Meeting . . . . . 451
State Board of Dental Examiners . . 500

T.

The Tooth Brush as a Prophylatic . . . 121 20
The Eyes and Teeth, Have they any Nerve Connection . . . . . 29
The Care of the Eyes in Children . . . 42
The Detection of Arsenic . . . . 47
The Digestive Movement in Plants . . 50
The Healing Element in Arnica . . . 51
The Preservation of the Teeth treated as a Specialty 77
The Relation of Dental Science to Medicine . 88
The Dental Luminary . . . . 104
The Teeth in Literature , . . 105
The Influence of Vicious Systemic States on the Teeth . . . . . 109
The Rubber Co. Again . . . . 180
The Skin and Bodily Temperature . . 225

The work of State Societies . . . 230
The Treatment of the Six Year Molars and the Separation of the Teeth . . . 249
Treatment of Stammering . . . 262
To Disguise Tannin . . . . 263
The M. V. A. and Yours Respectfully . . 281
The Lead Line of the Gums . . . 361
Tumors and Abscesses, Causes and Treatment , 413
Transplantation and Replantation of Teeth . 457
The Air an Anæsthetic . , . . 491
To Make Leaches Bite . . . . 498
Treatment of the Dental Pulp . . . 529

**V.**

Volume XXX. . . . . . 58

**W.**

Who Shall Confer Degrees . . . 49
Women at Michigan University . . . 98
Washington's Teeth . . , . 268

THE POCKET

### DENTAL REGISTER.

Every dentist with anything like a full practice, and who cares to conduct business in a systematic manner, must have a book in which to record his dental operations.

The large "Registers" that have been used for that purpose, are excellent in their place, and may still be used in connection with such a book as this, if desired; but they are open to several important objections.

Many dentists using them have doubtless experienced the inconvenience of going to the desk after each patient's sitting, to make a record, and have preferred trusting to memory till after office hours. By that time, the exact tooth operated upon has often been forgotten; and sometimes a certain operation has slipped from the mind altogether.

The fillings put in to-day are marked on the same diagram with those inserted a month or a year ago; consequently, after a little while, it is impossible to distinguish between old and recent work.

The space reserved for each account being so large, one is unwilling to spoil a blank to record trifling services, such as extracting or treating a tooth for a patient whom he may never see again; although it often happens afterward that he would be glad to have a knowledge of the service.

In recording operations on the temporary teeth, it is very unsatisfactory to cross out the twelve back teeth of the permanent set, and imagine the bicuspids to be temporary molars. In such cases, much confusion generally follows in the course of a few years.

This POCKET REGISTER is presented with the belief that it is free from these objectionable features.

Each operation may be recorded with pen or pencil, right at the chair, in almost the same time that an appointment is made for another sitting.

Every operation bears its own date, and cannot be taken for a previous or later one.

The space for accounts is so economically used, that one will not hesitate to record the most trivial services. It consists of 224 pages, arranged as follows:

120 pages for small accounts, ten to the page, with blanks for date, name, residence, operation, debit, and credit. Each account is also furnished with a diagram of the teeth for marking operations performed.
24 pages for medium accounts, two to the page, with blanks and diagram, as above.
16 pages for large accounts, one to the page, with blanks and diagram.
16 pages with blanks and diagrams expressly arranged for operations on the temporary teeth, ten accounts to the page.
32 pages for appointments for 64 weeks. These are without date, and may, therefore, be used for any time.
16 pages for index and memoranda.

It will be seen that the "Pocket Register" combines Account-book, Record of Operations on the Permanent and Temporary Teeth, Appointment-book, and Memoranda; and that the whole is offered for about the price of an ordinary Engagement-book.

It is printed on fine writing paper, bound handsomely in leather, with tuck, or in English cloth, and is of a suitable size for carrying in the breast-pocket.

— PRICE. —

*In Leather, with Tuck.* .......... $1 50

*In English Cloth,* .......... 1 25

Sent post-paid, on receipt of price. For sale, wholesale and retail, by

**For Sale by SPENCER, CROCKER & CO.**

AMERICAN DENTAL ASSOCIATION—Meets on the first Tuesday in Aug., 1870, at Philadelphia. *Pres.* A. L. Northrup, New York; *Cor. Sec.* J. H. McQuilien, Philadel'a; *Rec. Sec.* C. Stoddard Smith, Springfield, Ill.

*American Dental Convention* Meets at Philadelphia, second Tuesday in August, 1876. *Pres.* B. F. Coy, Baltimore; *Cor. Sec.* S. Welchens, Lancaster, Pa.; *Rec. Sec.* A. Tees, Philadelphia, Pa.

*List of Societies Represented in the American Dental Association.*

AMERICAN ACADEMY OF DENTAL SURGERY met in New York April 7, 1875. *Pres.* Geo. H. Perine; *Rec. Sec.* N. M. Beckwith; *Cor. Sec.* J. A. Bishop.

*American Academy of Dental Science* Meets at Boston, Mass. *Pres.* Joshua Tucker M. D., of Boston ; *Sec.* W. L. Tucker, of Boston ; *Cor. Sec.*, E. N. Harris.

*Brooklyn Dental Society* Meets semi-monthly. *Pres.* Wm. H. Atkinson; *Rec. Sec.* C. P. Crandell ; *Cor. Sec.* Wm. Fishbough.

*Buffalo Dental Society* Meets second Monday of each month at Buffalo. *Pres.* S. A. FREEMAN ; *Rec. Sec.* G. C. DABOLL.

*Boston Dental Institute*—Meets first Tuesday of each month. *Pres.* D. G. WILLIAMS, Buffalo; *Cor. Sec.* D. S. Dickerman.

*California State Dental Association* Meets annually in June. *Pres.* C. C. Knowles, San Francisco ; *Rec. Sec.* H. J. Plomteaux, Woodland.

*Chicago Dental Society* Meets monthly at Chicago. *Pres.* C. R. E. Koch; *Rec. Sec.* D. B. Freeman.

*Connecticut Valley Dental Association.* Meets semi-annually, second Tuesday of June and October. *Pres.* H. F. Bishop, Worcester, Mass.; *Rec. Sec.* C. T. Stockwell, Westfield, Mass. Next Meeting in Hartford. June, 1876.

*Connecticut State Dental Society. Pres.* R. C. Dunham, New Britain ; *Rec. Sec.*, D E. Lane, Hartford.

*Charleston Dental Association*—Meets second Tuesday of every month ; *Pres.* T. F. Chupein ; *Sec. and Treas.* B. A. Muckenfuss.

*Central Penn. Dental Association* Meets at Huntingdon, Jan., 1876. *Pres.* E. J. Green; *Sec.* W. B. Miller.

CINCINNATI DENTAL SOCIETY—Meets first and third Tuesday of each month. *Pres.* A. Berry; *Sec.* A. Downing.

*Delaware Dental Association*- -Meets semi-annually. *Pres.* E. W. Haines, Newark ; *Sec.* C. R. Jeffries, Wilmington.

*Dental Society of the State of Maryland and District of Columbia* Meets on Wednesday, Sept. 6, 1876, in Baltimore. *Pres.* R. B. Winder; *Rec. Sec.* R. F. Hunt; *Cor. Sec.* E. P. Keech.

*Eastern Indiana Dental Association.*—Meets semi-annually, first Tuesday in May and last Tuesday in October; *Pres.* J. K. Jameson, Connersville; *Sec.* J. W. White, Knightstown, Ind. (Place of meeting fixed by appointment.)

*Eastern Ohio Dental Association*- Meets on Tuesday, August 26th, 1873, at Alliance ; *Pres.* A. J. Doud. *Rec. Sec.* J. Porter.

*East Tennessee Dental Association*—Meets at Chattanooga, 3d Wednesday of Sept., 1876. *Pres.* S. M. Prothro; *Rec. Sec.*, S. B. Cooke.

*Georgia State Dental Society* Next meeting at Atlanta, 2d Tuesday in May, 1876. *Pres.* G. W. McEllaney; *Cor. Sec.* C. C. Allen, Marietta.

*Harris Dental Association* Meets quarterly on the first Thursday in February, May. August, Nov'ber. *Pres.* P. G. Hiestand, Millersville; *Sec.* W. N. Amer, Lancaster.

*Hudson Valley Dental Association* Meets monthly at Troy, N. Y. *Pres.* L. C. Wheeler, *Rec. Sec.* S. G. Andres ; *Cor. Sec.* S. D. French.

*Illinois State Dental Soeiety* Meets annually. *Pres.* E. D. Swain, Chicago; *Sec.* R. E. Coch; Chicago. Meets second Tuesday in May, 1876, at Galesburg.

*Indiana State Dental Society*—Meets next year at Indianapolis, on the first Tuesday of June. *Pres.* J. B. Clayton, Shelbyville; *Cor. and Rec. Sec.* P. McDonald, Goshen.

*Iowa State Dental Society*- -Meets annually. Next meeting at Burlington, on the second Tuesday of May. *Pres.* C. P. Artman Waterloo; *Rec. Sec.* R. L. Cochran Burlington; *Cor. Sec.* I. P. Wilson, Burlington.

*Kansas State Dental Society* Meets annually. Next meeting at Leavanworth, first Wednesday in May, 1875. *Pres.* L. C. Wasson; *Sec.* J. D Patterson.

*Kansas and Missouri Valley Dental Association*—Meets first and third Tuesdays of erch month. *Pres.* J. R. Stark ; *Sec.* H. C. Massie, Kansas City, Mo.

*Kentucky State Dental Association* Meets annually first Tuesday in June, next meeting in Louisville. *Pres.*, C. E Dunn; *Sec.*, A. C. Rawls.

*Lake Erie Dental Association* Meets Annually. *Pres.* C. B. Ansart, Oil City, Pa. *Sec.* C. D. Elliott, Franklin, Pa. Next meeting first Tuesday of May, 1876.

*Mad River Dental Association* Meets at Dayton on the first Tuesdays in April and October. *Pres.* S. B. Tizzard, Dayton ; *Sec.* A. J. Grosvenor, Troy.

*Maine Dental Society*—Meets in Aug., at Thomaston. *Pres.* E. J. Roberts, Augusta ; *Sec.* C. E. Hussey, Biddeford.

*Maryland Association of Dentists* Meets the last Thursday of every month. *Pres.* J. B. Bean ; *Sec.* S. M. Field.

*Massachusetts Dental Society*—Meets monthly. *Pres.* J. H. Batchelder, of Salem *MSec.* C. Wilson ; *Cor. Sec.* L. D. Shepard.

*Michigan State Dental Association*—Meets annually second Tuesday of Oct., at Grand Rapids. *Pres.* T. R. Perry, Grand Rapids; *Rec. & Cor. Sec.* Wm. Tremper, Ypsilanti

*Mississippi Valley Dental Association* Meets annually at Cincinnati, on first Wednesday in March, 1876. *Pres.* George Watt, Xenia, Ohio; *Rec. Sec.* Wm. Taft, Cin.

*Mississippi State Dental Association* will meet 3d Wednesday of Aug. '76. *Pres.* J. D. Miles; *Sec.* A. Riser.

*Minnesota Dental Associrtion* Meets annually. Next meeting at Minneapolis; May, 1876. *Pres.* F. A. Williamson, Reading, *Sec.* W. F. Lewis, Winona- St. Paul.

*Missouri Dental Association* Meets on the first Tuesday of June, in St. Louis. *Pres.* C. W. Rivers, St. Louis; *Sec.* W. H. Evans, St. Louis.

*Missouri Valley Dental Association* Meets on the fourth Tuesday in July, 1876, in Omaha, Neb. *Pres.* J. W. Chadduck; *Sec. and Treas.* F. M. Shrirer, Tabor, Iowa.

*Merrimac Valley Dental Association* Meets on the last Thursday of May, 1876, at Portland, Me. *Pres.* A. Dudley, Salem, Mass; *Rec. Sec.* W. E. Riggs, Lawrence, Mass., *Cor. Sec.* J. H. Kidder, Lawrence, Mass.

*Memphis Dental Society*—Meets on the first Tuesday of each month. *Pres.* W. M. Arrington; *Sec.* V. F. Elliott; *Treas.* C. L. Harris.

*Nashville Dental Association*—Meets on the second Tuesday of each month. *Pres.* J. C. Ross; *Sec.* R. R. Freeman; *Cor. Sec.* S. J. Cobb.

*New Jersey State Dental Society*—Meets annually. Next meeting at Atlantic City, on the second Tuesday in July, 1876. *Pres.* C. S. Stockton, Newark; *Sec.* C. A. Meeker, Newark.

*New Haven Dental Society*--Meets on the first Wednesday of each month at New Haven. *Pres.* J. T. Metcalf, *Rec and Cor. Sec.* C. L. Smith, of New Haven.

*New Orleans Dental Society* Meets monthly. *Pres.* ——; *Rec. Sec.* A. F. McLane.

*Northern Ohio Dental Association.* Meets annually, second Tuesday of June, 1876, at Cleveland. *Pres.* E. J. Way, Sandusky; *Cor. Sec.* C. Buffett, Clinton.

*Northern Iowa Dental Association*—Meets annually, on the second Tuesday in June, 1873. Next meeting at Waterloo; *Pres.* J. N. Abbott, Lancaster, *Rec. Sec.* A. V Eaton, Anamosa; *Cor. Sec.* A. R. Mason, Waterloo.

*North Carolina Deutal Association* Meets annually. Next meeting at Greensboro, 2d Tuesday of Aug., 1876. *Pres.* B. F. Arrington; *Sec.* E. L. Hunter.

*Ohio College Dental Association*--Meets annually Feb. 25. at Cincinnati. *Pres.* Jas. Leslie, Cin'ti.; *Rec. Sec.* H. A. Smith, of Cincinnati.

*Ohio State Dental Association*--Meets annually at Columbus. Next meeting, first Wednesday of December, 1876. *Pres.* C. R. Taft, Cincinnati; *Rec. Sec.* H. L Ambler, Cleveland, O.; *Cor. Sec.* A. F. Emminger, Columbus, O.

*Odontographic Society of Pennsylvania*--Meets on the first Wednesday of each month at 8 o'clock, P. M. in the Philadelphia Dental College. *Pres.* Louis Jack; *Cor. Sec.* J. H. McQuillen; *Rec. Sec.* E. L. Hewitt.

*Odontological Society of New York City* Meets the second Tuesday of each month. *Pres.* A. L. Northrup; *Rec. Sec.* Wm. Jarvie, Jr.

*Old Colony Dental Association* Meets quarterly.* *Pres.* C. G. Davis; *Rec. Sec.* L. W. Puffer, North Bridgewater, Mass; *Cor. Sec.* N. C. Fowler, Yarmouth, Mass.

*Ohio Valley Dental Association* Meets annually, in N. E. Ky; this year in Paris, Ky. Tuesday, Sept. 9th.

*Oregon State Dental Society* Meets annually. *Pres.* J. H. Hatch, Portland; *Rec. Sec.* J. R. Cardwell, Portland.

*Pennsylvania State Dental Society* meets next in Philadelphia, 2d Tuesday of July, 1876. *Pres.* E. T. Darby; *Rec. Sec.* R. H. Moffitt, Harrisburg; *Cor. Sec.* M. H. Webb, Lancaster.

*Pennsylvania Association of Dental Surgery* Meets monthly in Philadelphia. *Pres* Spencer Roberts; *Sec.* G. Pettit.

PITTSBURG DENTAL SOCIETY—Meets second Tuesday of each month. *Pres.* W. F. Fundenburg; *Sec.* M. L. King.

*San Francisco Dental Association* Meets monthly. *Pres.* C. C. Knowles; *Cor. Se* H. E. Knox; *Rec. Sec.* Wm. Dutch.

*St. Louis Dental Society* Meets monthly. *Pres.* W. H. Morrison; *Sec.* A. H. Fuller.

*Susquehanna Dental Association* Meets annually. *Pres.* W. H. Wissimer, Williamsport, Pa.; *Rec. Sec.* W H. Hertz. Meets May, 1876, at Pittston.

*Society of Dental Surgeons of the City of New York* Meets semi-monthly in New York. *Pres.* B. F. Franklin: *Rec. Sec.* J. S. Latimer; *Cor. Sec.* E. H. Burras.

**Southern Dental Association* Meets annually. Next meeting at Memphis, Tenn., at time of Mardi-Gras. *Pres.* J. R. Walker, New Orleans, La.; *Rec. Sec.* J. F Thompson Va

*South Carolina State Dental Association* Meets annually. *Pres.* G. F. S. Wright, Columbia; *Sec.* F. T. Chupein, Charleston; *Cor. Sec.* J. S. Thompson, Abbeville. Next meeting at Greenville, S. C., second Thursday of June, 1876.

*Tennessee Dental Association* Meets semi-annually. Next meeting in Nashville, on the 4th Wednesday in June. 1876. *Pres.* R. R. Freeman; *Rec. Sec.* W. H. Morgan. *Cor. Sec.* L. G. Noel.

*Virginia State Dental Association* Meets in Richmond, first Thursday in Nov, of each year. *Pres.* H. M. Grant, Abington: *Sec.* G. F. Keesee, Richmond.

*Washington, City Dental Society* Meets annually. *Pres.*, R. F. Hunt, *Sec.* Dr. Evans,

*Wisconsin State Dental Association* Meets annually. Next meeting at Milwaukee, second Tuesday of July, 1876. *Pres.* R. S. Wells. Sparta; M. T. Moore, La Crosse.

### State and District Dental Societies of State of New York.

*New York State Dental Society*--Meets at Albany on the second Wednesday of July next. *Pres.* W. C. Barrett, Warsaw, N. Y.; *Cor. Sec.* S. B. Palmer; *Rec. Sec.* S. S. A, Freeman, Buffalo.

*First District* Meets second and fourth Wednesday evenings of each month. Annual meeting in New York, first Tuesday in June. *Pres.* J. S. Latimer New York. *Rec. Sec.* F. M. Odell, New York.

*Second District.* Annual meeting in Brooklyn, first Monday in April. *Pres.* W. S. Elliott; *Rec. Sec.* M, E. Elmandorf. Brooklyn; *Cor. Sec.* F. W. Dalbare,

*Third District.* Meets semi-annually on second Tuesday in January and July. Annual meeting at Albany on second Tuesday in January. *Pres.* S. P. Welsh; *Rec. Sec.* H. A. Hall, Troy; *Cor, Sec.* J. A. Perkins, Albany.

*Fourth District.* Meets semi-annually. Meets annually at Saratoga on second Tuesday in June. *Pres.* C, A. Elmore, Ft. Edward; *Rec. Sec.* E. S. Pearsall, Saratoga.

*Fifth District.* Meets semi-annually. Annual meeting at Syracuse. second Tuesday in June. *Pres.* J. D. Huntington, Watertown; *Rec. Sec.* F. D. Nellis, Syracuse; *Cor. Sec.* J. S. Marshall, Syracuse.

*Sixth District.* Meets semi-annually. Annual meeting at Binghampton on second Tuesday in June. *Pres.* Frank B. Darby, Oswego; *Rec. Sec.* F. E. Perry, Norwich; *Cor. Sec.* A. M. Holmes, Morrisville.

*Seventh District.* Meets semi-annually. Annual meeting at Rochester first Tuesday in June. *Pres.* H. S. Miller; *Rec. Sec.* J. E. Line, Rochester.

*Eighth District.* Meets semi-annually. Annual on second Tuesday in May. *Pres* G. C. Daboll, *Rec. Sec.* S: A. Freeman, *Cor. Sec.* T. G. Lewis, Buffalo.

*The Seventh and Eighth District Societies* hold a union meeting on the last Tuesday of October. alternately at Buffalo and Rochester.

The Ohio State Board of Dental Examiners will meet in Cincinnati on the first Wednesday of March 1876, at 12 o'clock. *Pres.* J. Taft; *Sec.* W. P. Horton.

### Exchanges.

ST. LOUIS MEDICAL AND SURGICAL JOURNAL.
BOSTON MEDICAL AND SURGICAL JOURNAL.
THE PACIFIC MEDICAL AND SURGICAL JOURNAL, SAN FRANCISCO.
PHIDADELPHIA MEDICAL AND SURGICAL JOURNAL.
CINCINNATI MEDICAL ADVANCE.
CINCINNATI LANCET AND OBSERVER.
DENTAL COSMOS, PHILADELPHIA,
LADIES' REPOSITORY, CINCINNATI.
HALL'S JOURNAL OF HEALTH, N. Y.
CHICAGO MEDICAL EXAMINER,
THE DETROIT REVIEW OF MEDICINE AND PHARMACY.
MEDICAL RECORD, N. Y.
NASHVILLE JOURNAL OF MEDICINE AND SURGERY.
AMERICAN JOURNAL OF DENTAL SCIENCE, BALTIMORE.
THE UNITED STATES MEDICAL AND SURGICAL JOURNAL, CHICAGO.
NEW ORLEANS JOURNAL OF MEDICINE,
AMERICAN AGRICULTURIST, N. Y.
BOSTON JOURNAL OF CHEMISTRY.
JOURNAL OF APPLIED CHEMISTRY, N. Y.
CANADA JOURNAL OF DENTAL SCIENCE, MONTREAL.
INDIANA JOURNAL OF MEDICINE,
MEDICAL AND SURGICAL REPORTER, SALEM, OREGON.
MISSOURI DENTAL JOURNAL, ST. LOUIS.
ECLECTIC MEDICAL JOURNAL, CINCINNATI.

### HARVARD UNIVERSITY.

### Dental Department.

### BOSTON, MASS. 1875–76.

#### FACULTY.

Charles William Eliot, L.L.D., *President.*
Oliver W. Holmes, M.D., Professor of Anatomy.
Henry J. Bigelow, M.D., Professor of Surgery and Clinical Surgery.
Thomas H. Chandler, D.M.D., Professor of Mechanical Dentistry.
George T. Moffatt, M.D., D.M.D., Professor of Operative Dentistry.
Luther D. Shepard, D.D.S., Adjunct Professor of Operative Dentistry.
Nathaniel W. Hawes, Assistant Professor of Operative Dentistry.
Edward S. Wood, M.D., Assistant Professor of Chemistry.
Henry P. Bowditch, M.D., Assistant Professor of Physiology.
William H. Rollins, D.M.D., Instructor in Dental Pathology.
Charles A. Brackett, D.M.D., Instructor in Dental Therapeutics.
Ira A. Salmon, D.D.S., University Lecturer on Operative Dentistry.
Charles B. Porter, M.D., Demonstrator of Practical Anatomy.
Charles Wilson, D.M.D., Demonstrator in Charge.
George F. Grant, D.M.D., Demonstrator of Mechanical Dentistry.

The Ninth year of this School begins on the 28th of September and continues till the last Wednesday in June. The distinction of Summer and Winter sessions being hereafter abolished. It will be divided into two equal terms, with a recess of one week at Christmas. The course of instruction will be progressive, extending over two years, the lectures of one year not being repeated in the next.

The regular examinations will be held in the following order, viz.: At the end of the first year, anatomy, including dissection, physiology and general chemistry. A certificate from the demonstrator of anatomy will be required of each student, that he has satisfactorily dissected the three parts of the body.

At the end of the second year, dental pathology, including a knowledge of gestation and diseases of women so far as they affect the mouth and throat, dental materia medica and therapeutics, oral surgery and surgical pathology, operative and mechanical dentistry. The examinations in operative and mechanical dentistry will include actual operations and the preparation of specimens of mechanical dentistry.

Every candidate must be twenty-one years of age, and of good moral character; he must give evidence of having studied medicine or dentistry three full years; he must have spent at least one continuous year at this school, have presented a satisfactory thesis and have passed all the required examinations.

The University Degree, D.M.D. (*Dentariæ Medicinæ Doctor*), is conferred upon those who fulfil the requirements.

Students may be admitted to advanced standing upon passing a satisfactory examination in a majority of the studies already pursued by the class; but no student shall advance with his class or be admitted to advance standing until he has passed such examination.

The work in the operative and mechanical infirmaries will go on throughout the course, but no student shall be permitted to operate at the chair until he has by observation and practice on extracted teeth satisfied the Professor of his fitness.

The faculty recommend young men who propose to take the degree to spend the whole of the required term of three years of study in the school. But those who wish to spend but two of the three years in the school are earnestly advised to pass their first year of study, before entering, under the direction of a *competent* private instructor.

Unsurpassed facilities for operative and mechanical practice are furnished by the infirmary of the Massachusetts General Hospital which is open throughout the year.

#### FEES.

There are no fees for matriculation, for the diploma, nor for the demonstrators. For the first year a student is a member of the school, the fee is $200; for the second year, $150; for any subsequent year, $50.

For further information address,

**THOS. H. CHANDLER, Dean,**

**222 Tremont Street, Boston, Mass.**

AMERICAN DENTAL ASSOCIATION—Meets on the first Tuesday in Aug., 1870, at Philadelphia. *Pres.* A. L. Northrup, New York; *Cor. Sec.* J. H. McQuilien, Philadel'a; *Rec. Sec.* C. Stoddard Smith, Springfield, Ill.

*American Dental Convention* Meets at Philadelphia, second Tuesday in August, 1876. *Pres.* B. F. Coy, Baltimore; *Cor. Sec.* S. Welchens, Lancaster, Pa.; *Rec. Sec.* A. Tees, Philadelphia, Pa.

*List of Societies Represented in the American Dental Association.*

AMERICAN ACADEMY OF DENTAL SURGERY met in New York April 7, 1875. *Pres.* Geo. H. Perine; *Rec. Sec.* N. M. Beckwith; *Cor. Sec.* J. A. Bishop.

*American Academy of Dental Science* Meets at Boston, Mass. *Pres.* Joshua Tucker M. D., of Boston; *Sec.* W. L. Tucker, of Boston; *Cor. Sec.*, E. N. Harris.

*Brooklyn Dental Society* Meets semi-monthly. *Pres.* Wm. H. Atkinson; *Rec. Sec.* C. P. Crandell; *Cor. Sec.* Wm. Fishbough.

*Buffalo Dental Society* Meets second Monday of each month at Buffalo. *Pres.* S. A. FREEMAN; *Rec. Sec.* G. C. DABOLL.

*Boston Dental Institute*—Meets first Tuesday of each month. *Pres.* D. G. WILLIAMS, Buffalo; *Cor. Sec.* D. S. Dickerman.

*California State Dental Association* Meets annually in June. *Pres.* C. C. Knowles, San Francisco; *Rec. Sec.* H. J. Plomteaux, Woodland.

*Chicago Dental Society* Meets monthly at Chicago. *Pres.* C. R. E. Koch; *Rec. Sec.* D. B. Freeman.

*Connecticut Valley Dental Association.* Meets semi-annually, second Tuesday of June and October. *Pres.* H. F. Bishop, Worcester, Mass.; *Rec. Sec.* C. T. Stockwell, Westfield, Mass. Next Meeting in Hartford, June, 1876.

*Connecticut State Dental Society.* *Pres.* R. C. Dunham, New Britain; *Rec. Sec.*, D. E. Lane, Hartford.

*Charleston Dental Association*—Meets second Tuesday of every month; *Pres.* T. F. Chupein; *Sec. and Treas.* B. A. Muckenfuss.

*Central Penn. Dental Association* Meets at Huntingdon, Jan., 1876. *Pres.* E. J. Green; *Sec.* W. B. Miller.

CINCINNATI DENTAL SOCIETY—Meets first and third Tuesday of each month. *Pres.* A. Berry; *Sec.* A. Downing.

*Delaware Dental Association*—Meets semi-annually. *Pres.* E. W. Haines, Newark; *Sec.* C. R. Jeffries, Wilmington.

*Dental Society of the State of Maryland and District of Columbia* Meets on Wednesday, Sept. 6, 1876, in Baltimore. *Pres.* R. B. Winder; *Rec. Sec.* R. F. Hunt; *Cor. Sec.* E. P. Keech.

*Eastern Indiana Dental Association.*—Meets semi-annually, first Tuesday in May and last Tuesday in October; *Pres.* J. K. Jameson, Connersville; *Sec.* J. W. White, Knightstown, Ind. (Place of meeting fixed by appointment.)

*Eastern Ohio Dental Association*—Meets on Tuesday, August 26th, 1873, at Alliance; *Pres.* A. J. Doud. *Rec. Sec.* J. Porter.

*East Tennessee Dental Association*—Meets at Chattanooga, 3d Wednesday of Sept., 1876. *Pres.* S. M. Prothro; *Rec. Sec.*, S. B. Cooke.

*Georgia State Dental Society* Next meeting at Atlanta, 2d Tuesday in May, 1876. *Pres.* G. W. McEllaney; *Cor. Sec.* C. C. Allen, Marietta.

*Harris Dental Association* Meets quarterly on the first Thursday in February, May, August, Nov'ber. *Pres.* P. G. Hiestand, Millersville; *Sec.* W. N. Amer, Lancaster.

*Hudson Valley Dental Association* Meets monthly at Troy, N. Y. *Pres.* L. C. Wheeler, *Rec. Sec.* S. G. Andres; *Cor. Sec.* S. D. French.

*Illinois State Dental Soeiety* Meets annually. *Pres.* E. D. Swain, Chicago; *Sec.* R. E. Coch; Chicago. Meets second Tuesday in May, 1876, at Galesburg.

*Indiana State Dental Society*—Meets next year at Indianapolis, on the last Tuesday of June, 1876. *Pres.* J. K. Jameson, Connersville; *Sec.* J. B. Clayton, Shelbyville.

*Iowa State Dental Society*—Meets annually. Next meeting at Burlington, on the second Tuesday of May. *Pres.* C. P. Artman Waterloo; *Rec. Sec.* R. L. Cochran Burlington; *Cor. Sec.* I. P. Wilson, Burlington.

*Kansas State Dental Society* Meets annually. Next meeting at Leavanworth, first Wednesday in May, 1875. *Pres.* L. C. Wasson; *Sec.* J. D Patterson.

*Kansas and Missouri Valley Dental Association*—Meets first and third Tuesdays of erch month. *Pres.* J. R. Stark; *Sec.* H. C. Massie, Kansas City, Mo.

*Kentucky State Dental Association* Meets annually first Tuesday in June, next meeting in Louisville. *Pres.*, C. E Dunn; *Sec.*, A. C. Rawls.

*Lake Erie Dental Association* Meets Annually. *Pres.* C. B. Ansart, Oil City, Pa. *Sec.* C. D. Elliott, Franklin, Pa. Next meeting first Tuesday of May, 1876.

*Mad River Dental Association* Meets at Dayton on the first Tuesdays in April and October. *Pres.* R. Corson, Middletown; *Sec.* A. J. Grosvenor, Troy.

*Maine Dental Society*—Meets in Aug., at Thomaston. *Pres.* E. J. Roberts, Augusta; *Sec.* C. E. Hussey, Biddeford.

*Maryland Association of Dentists* Meets the last Thursday of every month. *Pres.* J. B. Bean; *Sec.* S. M. Field.

*Massachusetts Dental Society*—Meets monthly. *Pres.* John T. Codman, Boston; *Rec. Sec.* G. F. Grant, Boston; *Cor. Sec.* T. N, Chandler, Boston.

*Michigan State Dental Association*—Meets annually second Tuesday of Oct., at Grand Rapids. *Pres.* J. W. Finch, Adrian; *Rec. & Cor. Sec.* W. P. Morgan, Saginaw.

*Mississippi Valley Dental Association* Meets annually at Cincinnati, on first Wednesday in March, 1876. *Pres.* George Watt, Xenia, Ohio; *Rec. Sec.* Wm. Taft, Cin.

*Mississippi State Dental Association* will meet 3d Wednesday of Aug. '76. *Pres.* J. D. Miles; *Sec.* A. Riser.

*Minnesota Dental Associrtion* Meets annually. Next meeting at Minneapolis; May, 1876. *Pres.* F. A. Williamson, Reading, *Sec.* W. F. Lewis, Winona-St. Paul.

*Missouri Dental Association* Meets on the first Tuesday of June, in St. Louis. *Pres.* C. W. Rivers, St. Louis; *Sec.* W. H. Evans, St. Louis.

*Missouri Valley Dental Association* Meets on the fourth Tuesday in July, 1876, in Omaha, Neb. *Pres.* J. W. Chadduck; *Sec. and Treas.* F. M. Shrirer, Tabor, Iowa.

*Merrimac Valley Dental Association* Meets on the last Thursday of May, 1876, at Portland, Me. *Pres.* A. Dudley, Salem, Mass; *Rec. Sec.* W. E. Riggs, Lawrence, Mass., *Cor. Sec.* J. H. Kidder, Lawrence, Mass.

*Memphis Dental Society*—Meets on the first Tuesday of each month. *Pres.* W. M. Arrington ; *Sec.* V. F. Elliott ; *Treas.* C. L. Harris.

*Nashville Dental Association*—Meets on the second Tuesday of each month. *Pres.* J. C. Ross ; *Sec.* R. R. Freeman ; *Cor. Sec.* S. J. Cobb.

*New Jersey State Dental Society*—Meets annually. Next meeting at Atlantic City, on the second Tuesday in July, 1876. *Pres.* C. S. Stockton, Newark; *Sec.* C. A. Meeker, Newark.

*New Haven Dental Society*--Meets on the first Wednesday of each month at New Haven. *Pres.* J. T. Metcalf, *Rec and Cor. Sec.* C. L. Smith, of New Haven.

*New Orleans Dental Society* Meets monthly. *Pres.* ———; *Rec. Sec.* A. F. McLane.

*Northern Ohio Dental Association.* Meets annually, second Tuesday of June, 1876, a t Cleveland. *Pres.* E. J. Way, Sandusky; *Cor. Sec.* C. Buffett, Clinton.

*Northern Iowa Dental Association*—Meets annually, on the second Tuesday in June, 1873. Next meeting at Waterloo ; *Pres.* J. N. Abbott, Lancaster, *Rec. Sec.* A. V Eaton, Anamosa ; *Cor. Sec.* A. R. Mason, Waterloo.

*North Carolina Deutal Association* Meets annually. Next meeting at Greensboro, 2d Tuesday of Aug., 1876. *Pres.* B. F. Arrington; *Sec.* E. L. Hunter.

*Ohio College Dental Association*—Meets annually Feb. 25. at Cincinnati. *Pres.* Jas. Leslie, Cin'ti.; *Rec. Sec.* H. A. Smith, of Cincinnati.

*Ohio State Dental Association*--Meets annually at Columbus. Next meeting, first Wednesday of December, 1876. *Pres.* C. R. Taft, Cincinnati; *Rec. Sec.* H. L Ambler, Cleveland, O.; *Cor. Sec.* A. F. Emminger, Columbus, O.

*Odontographic Society of Pennsylvania*--Meets on the first Wednesday of each month at 8 o'clock, P. M. in the Philadelphia Dental College. *Pres.* Louis Jack; *Cor. Sec.* J. H. McQuillen; *Rec. Sec.* E. L. Hewitt.

*Odontological Society of New York City* Meets the second Tuesday of each month. *Pres.* A. L. Northrup ; *Rec. Sec.* Wm. Jarvie, Jr.

*Old Colony Dental Association* Meets quarterly.* *Pres.* C. G. Davis ; *Rec. Sec.* L. W. Puffer, North Bridgewater, Mass ; *Cor. Sec.* N. C. Fowler, Yarmouth, Mass.

*Ohio Valley Dental Association* Meets annually, in N. E. Ky ; this year in Paris, Ky. Tuesday, Sept. 9th.

*Oregon State Dental Society* Meets annually. *Pres.* J. H. Hatch, Portland ; *Rec. Sec.* J. R. Cardwell, Portland.

*Pennsylvania State Dental Society* meets next in Philadelphia, 2d Tuesday of July, 1876 *Pres.* E. T. Darby; *Rec. Sec.* R. H. Moffitt, Harrisburg; *Cor. Sec.* M. H. Webb, Lancaster.

*Pennsylvania Association of Dental Surgery* Meets monthly in Philadelphia. *Pres* Spencer Roberts; *Sec.* G. Pettit.

PITTSBURG DENTAL SOCIETY—Meets second Tuesday of each month. *Pres.* W. F. Fundenburg; *Sec.* M. L. King.

*San Francisco Dental Association* Meets monthly. *Pres.* C. C. Knowles ; *Cor. Se* H. E. Knox ; *Rec. Sec.* Wm. Dutch.

*St. Louis Dental Society* Meets monthly. *Pres.* W. H. Morrison ; *Sec.* A. H. Fuller.

*Susquehanna Dental Association* Meets annually. *Pres.* W. H. Wissimer, Williamsport, Pa.; *Rec. Sec.* W H. Hertz. Meets May, 1876, at Pittston.

*Society of Dental Surgeons of the City of New York* Meets semi-monthly in New York. *Pres.* B. F. Franklin : *Rec. Sec.* J. S. Latimer ; *Cor. Sec.* E. H. Burras.

**Southern Dental Association* Meets annually. Next meeting at Memphis, Tenn., at time of Mardi-Gras. *Pres.* J. R. Walker, New Orleans, La.; *Rec. Sec.* J. F Thompson Va.

*South Carolina State Dental Association* Meets annually. *Pres.* G. F. S. Wright, Columbia; *Sec.* F. T. Chupein, Charleston; *Cor. Sec.* J. S. Thompson, Abbeville. Next meeting at Greenville, S. C., second Thursday of June, 1876.

*Tennessee Dental Association* Meets semi-annually. Next meeting in Nashville, on the 4th Wednesday in June. 1876. *Pres.* R. R. Freeman; *Rec. Sec.* W. H. Morgan *Cor. Sec.* L. G. Noel.

*Virginia State Dental Association* Meets in Richmond, first Thursday in Nov. of each year. *Pres.* H. M. Grant, Abington : *Sec.* G. F. Keesee, Richmond.

*Washington, City Dental Society* Meets annually. *Pres.*, R. F. Hunt, *Sec.* Dr. Evans.

*Wisconsin State Dental Association* Meets annually. Next meeting at Milwaukee, second Tuesday of July, 1876. *Pres.* R. S. Wells, Sparta; M. T. Moore, La Crosse.

### State and District Dental Societies of State of New York.

*New York State Dental Society*--Meets at Albany on the second Wednesday of July next. *Pres.* W. C. Barrett, Warsaw, N. Y.; *Cor. Sec.* S. B. Palmer; *Rec. Sec.* S. S. A, Freeman, Buffalo.

*First District* Meets second and fourth Wednesday evenings of each month. Annual meeting in New York, first Tuesday in June. *Pres.* J. S. Latimer New York. *Rec. Sec.* F. M. Odell, New York.

*Second District.* Annual meeting in Brooklyn, first Monday in April. *Pres.* W. S. Elliott; *Rec. Sec.* M. E. Elmandorf. Brooklyn; *Cor. Sec.* F. W. Dalbare.

*Third District.* Meets semi-annually on second Tuesday in January and July. Annua meeting at Albany on second Tuesday in January. *Pres.* S. P. Welsh; *Rec. Sec.* H A. Hall, Troy; *Cor. Sec.* J. A. Perkins, Albany.

*Fourth District.* Meets semi-annually. Meets annually at Saratoga on second Tuesday in June. *Pres.* C, A. Elmore, Ft. Edward; *Rec. Sec.* E. S. Pearsall, Saratoga.

*Fifth District.* Meets semi-annually. Annual meeting at Syracuse. second Tuesday in June. *Pres.* J. D. Huntington, Watertown; *Rec. Sec.* F. D. Nellis, Syracuse; *Cor. Sec.* J. S. Marshall, Syracuse.

*Sixth District.* Meets semi-annually. Annual meeting at Binghampton on second Tuesday in June. *Pres.* Frank B. Darby, Oswego; *Rec. Sec.* F. E. Perry, Norwich; *Cor. Sec.* A. M. Holmes, Morrisville.

*Seventh District.* Meets semi-annually. Annual meeting at Rochester first Tuesday in June. *Pres.* H. S. Miller; *Rec. Sec.* J. E. Line, Rochester.

*Eighth District.* Meets semi-annually. Annual on second Tuesday in May. *Pres* G. C. Daboll, *Rec. Sec.* S. A. Freeman, *Cor. Sec.* T. G. Lewis, Buffalo.

*The Seventh and Eighth District Societies* hold a union meeting on the last Tuesday of October. alternately at Buffalo and Rochester.

The Ohio State Board of Dental Examiners will meet in Cincinnati on the first Wednesday of March 1876, at 12 o'clock. *Pres.* J. Taft; *Sec.* W. P. Horton.

### Exchanges.

ST. LOUIS MEDICAL AND SURGICAL JOURNAL.
BOSTON MEDICAL AND SURGICAL JOURNAL.
THE PACIFIC MEDICAL AND SURGICAL JOURNAL, SAN FRANCISCO.
PHIDADELPHIA MEDICAL AND SURGICAL JOURNAL.
CINCINNATI MEDICAL ADVANCE.
CINCINNATI LANCET AND OBSERVER.
DENTAL COSMOS, PHILADELPHIA,
LADIES' REPOSITORY, CINCINNATI.
HALL'S JOURNAL OF HEALTH, N. Y.
CHICAGO MEDICAL EXAMINER,
THE DETROIT REVIEW OF MEDICINE AND PHARMACY.
MEDICAL RECORD, N. Y.
NASHVILLE JOURNAL OF MEDICINE AND SURGERY.
AMERICAN JOURNAL OF DENTAL SCIENCE, BALTIMORE.
THE UNITED STATES MEDICAL AND SURGICAL JOURNAL, CHICAGO.
NEW ORLEANS JOURNAL OF MEDICINE,
AMERICAN AGRICULTURIST, N. Y.
BOSTON JOURNAL OF CHEMISTRY.
JOURNAL OF APPLIED CHEMISTRY, N. Y.
CANADA JOURNAL OF DENTAL SCIENCE, MONTREAL.
INDIANA JOURNAL OF MEDICINE,
MEDICAL AND SURGICAL REPORTER, SALEM, OREGON.
MISSOURI DENTAL JOURNAL, ST. LOUIS.
ECLECTIC MEDICAL JOURNAL, CINCINNATI.

### HARVARD UNIVERSITY.

### Dental Department.

### BOSTON, MASS. 1875–76.

---

**FACULTY.**

Charles William Eliot, L.L.D., *President.*
Oliver W. Holmes, M.D., Professor of Anatomy.
Henry J. Bigelow, M.D., Professor of Surgery and Clinical Surgery.
Thomas H. Chandler, D.M.D., Professor of Mechanical Dentistry.
George T. Moffatt, M.D., D.M.D., Professor of Operative Dentistry.
Luther D. Shepard, D.D.S., Adjunct Professor of Operative Dentistry.
Nathaniel W. Hawes, Assistant Professor of Operative Dentistry.
Edward S. Wood, M.D., Assistant Professor of Chemistry.
Henry P. Bowditch, M.D., Assistant Professor of Physiology.
William H. Rollins, D.M.D., Instructor in Dental Pathology.
Charles A. Brackett, D.M.D., Instructor in Dental Therapeutics.
Ira A. Salmon, D.D.S., University Lecturer on Operative Dentistry.
Charles B. Porter, M.D., Demonstrator of Practical Anatomy.
Charles Wilson, D.M.D., Demonstrator in Charge.
George F. Grant, D.M.D., Demonstrator of Mechanical Dentistry.

---

The Ninth year of this School begins on the 28th of September and continues till the last Wednesday in June. The distinction of Summer and Winter sessions being hereafter abolished. It will be divided into two equal terms, with a recess of one week at Christmas. The course of instruction will be progressive, extending over two years, the lectures of one year not being repeated in the next.

The regular examinations will be held in the following order, viz.: At the end of the first year, anatomy, including dissection, physiology and general chemistry. A certificate from the demonstrator of anatomy will be required of each student, that he has satisfactorily dissected the three parts of the body.

At the end of the second year, dental pathology, including a knowledge of gestation and diseases of women so far as they affect the mouth and throat, dental materia medica and therapeutics, oral surgery and surgical pathology, operative and mechanical dentistry. The examinations in operative and mechanical dentistry will include actual operations and the preparation of specimens of mechanical dentistry.

Every candidate must be twenty-one years of age, and of good moral character; he must give evidence of having studied medicine or dentistry three full years; he must have spent at least one continuous year at this school, have presented a satisfactory thesis and have passed all the required examinations.

The University Degree, D.M.D. (*Dentariæ Medicinæ Doctor*), is conferred upon those who fulfil the requirements.

Students may be admitted to advanced standing upon passing a satisfactory examination in a majority of the studies already pursued by the class; but no student shall advance with his class or be admitted to advance standing until he has passed such examination.

The work in the operative and mechanical infirmaries will go on throughout the course, but no student shall be permitted to operate at the chair until he has by observation and practice on extracted teeth satisfied the Professor of his fitness.

The faculty recommend young men who propose to take the degree to spend the whole of the required term of three years of study in the school. But those who wish to spend but two of the three years in the school are earnestly advised to pass their first year of study, before entering, under the direction of a *competent* private instructor.

Unsurpassed facilities for operative and mechanical practice are furnished by the infirmary of the Massachusetts General Hospital which is open throughout the year.

---

**FEES.**

There are no fees for matriculation, for the diploma, nor for the demonstrators. For the first year a student is a member of the school, the fee is $200; for the second year, $150; for any subsequent year, $50.

For further information address,

**THOS. H. CHANDLER, Dean,**

**222 Tremont Street, Boston, Mass.**

### OHIO COLLEGE
### OF
### DENTAL SURGERY.

The THIRTY-FIRST ANNUAL SESSION of the OHIO COLLEGE OF DENTAL SURGERY will begin on the 18th of October, 1876, and continue till March following.

### FACULTY.

JAMES TAYLOR, M. D., D. D. S., Emeritus Institutes of Dental Science.
WM. CLENDENIN, M. D., Anatomy.
J. L, CILLEY, M. D., Pyhsiology and Histology.
F. BRUNNING, M. D., Pathology and Therapeutics.
J. TAFT, D. D. S., Operative Dentistry, Hygiene, Microscopy.
J. S. CASSIDY, D. D. S., Chemistry.
H. M. REID, Clinical Dentistry.
WM. VAN ANTWERP, D. D. S., Mechanical Dentistry.
J. L. CILLEY, M. D., Practical Anatomy

### SPECIAL PROFESSORS.

GEO. WATT, M. D., D. D. S.,Diseases Women and Children with reference to Dentistry.
F. H. REHWINKEL, M. D., D. D. S., Oral Surgery.

| | |
|---|---|
| Matriculation Fee (but once)............... | $ 5.00 |
| Professors' Tickets for one session.......... | 100.00 |
| Or for Winter and Spring terms.............. | 130.00 |
| Demonstrator's Ticket (for Anatomy)...... | 5.00 |
| Diploma Fee........................................ | 30.00 |

### TEXT BOOKS.

Gray's or Wilson's Anatomy; Dalton's, Carpenter's or Kirk's Phy sio logy; U. S. Dispensatory; Fowne's, Turner's or Graham's Chemistry: Watt's Chemical Essays; Taft's Operative Dentistry; Richardson's Mechanical Dentistry; Harris' Principles and Practice of Dental Science; Tome's Dental Surgery; Harris' Dictionary of Dental Surgery,

### TERMS OF GRADUATION.

A canditate for gradustion must have two full years of pupilage, part of which, at least, should be with a reputable dental practitioner, and good teacher, inclusive of two complete courses of lectures in a Dental College.

A graduate of a respectable Medical College who has had one year's pupilage under a reputable dentist, or a student having had a regular pupilage of two years and four years practice, and otherwise satisfactory, may be admitted to the Senior Class, and to examination for the degree of D. D. S., after attending one full course of lectures.

For further particulars address the Dean or Secretary by letter.

Students coming to the city should call on the Dean. Good board can be had for $4.00 to $7.00 a week.

### SPRING COURSE, 1877.

The Spring course in this Institution will commence on the 15th of March and continue till the 1st of June.

Tickets for the course, $50, to be paid on entering; or $130 for Spring and Winter term. tf

F. BRUNNING, SEC. J. TAFT, DEAN, 117 West 4th St.

### The Dental College

OF THE

### UNIVERSITY of MICHIGAN

The Second Annual Session of this Institution will commence on the 1st of October and close on the last Wednesday of March, thus making a course of six months. The regular course of instruction commences at once and will proceed through the term with the customary holiday vacation.

The students in this department will receive instructions in Anatomy, Physiology, Pathology, Chemistry, Materia Medica, Therapeutics and Surgery, from the professors of their respective branches in the *Department of Medicine* and Surgery of the University, when lectures commence and continue the same as with the Dental College.

### FACULTY OF THE DENTAL DEPARTMENT.

J. B. Angell, LL. D., President.

J. Taft, D. D. S., Professor of Principles aud Practice of Operative Dentistry.

J. A. Watling, D. D, S., Professor of Clinical and Mechanical Dentistry.

W. H. Jackson, Demonstrator.

It is desirable that students should be present at the opening of the session, as the regular course of instruction begins at that time. Seats in the lecture-room are assigned by selection to students in the order of registration on the steward's books; and each student is expected to occupy during the session such seat as he may select. Students on arriving in Ann Arbor should call at the steward's office.

### CONDITION OF GRADUATION.

The candidate must be twenty-one years of age. He must furnish evidence of good moral character.

He must devote three years in the study of his profession, in connection with attendance upon a course of medical lectures. He must attend two full courses of lectures in the Dental College, or one course elsewhere and the last one here, and we recommend that he attend three courses regularly.

He must sustain an examination satisfactory to the Faculty in all the branches taught.

A graduate of the Medical College may enter the senior class, and, if found qualified, may graduate after one year has been devoted to the study of dentistry.

Fees and Expenses.—The fees which must be paid in advance, are as follows:

Matriculation Fee.—Residents of Michigan, $10.00; non-residents, $15.00.

Annual Dues.—Residents of Mcchigan, $15.00; non-residents, $20.00.

Graduation Fee.—For all alike $5.00. The admission fee is paid, but once, and entitles the students to the privileges of permanent membership in any department of the University. The annual tax is paid the first year and every year after.

For further particulars address the Dean of the Dental College, Ann Arbor, Michigan.

tf J. TAFT, Dean.

### OHIO COLLEGE
OF
### DENTAL SURGERY.

The Thirty-First Annual Session of the Ohio College of Dental Surgery will begin on the 18th of October, 1876, and continue till March following.

### FACULTY.

James Taylor, M. D., D. D. S., Emeritus Institutes of Dental Science.
Wm. Clendenin, M. D., Anatomy.
J. L. Cilley, M. D., Physiology and Histology.
F. Brunning, M. D., Pathology and Therapeutics.
J. Taft, D. D. S., Operative Dentistry, Hygiene, Microscopy.
J. S. Cassidy, D. D. S., Chemistry.
H. M. Reid, Clinical Dentistry.
Wm. Van Antwerp, D. D. S., Mechanical Dentistry.
J. L. Cilley, M. D., Practical Anatomy

### SPECIAL PROFESSORS.

Geo. Watt, M. D., D. D. S., Diseases of Women and Children with reference to Dentistry.
F. H. Rehwinkel, M. D., D. D. S., Oral Surgery.

### FEES.

| | |
|---|---|
| Matriculation Fee (but once) | $ 5.00 |
| Professors' Tickets for one session | 100.00 |
| Or for Winter and Spring terms | 130.00 |
| Demonstrator's Ticket (for Anatomy) | 5.00 |
| Diploma Fee | 30.00 |

### TEXT BOOKS.

Gray's or Wilson's Anatomy; Dalton's, Carpenter's or Kirk's Physiology; Wagner's Pathology; U. S. Dispensatory; Fowne's, Turner's or Graham's Chemistry: Watt's Chemical Essays; Taft's Operative Denistry; Richardson's Mechanical Dentistry; Harris' Principles and Practice of Dental Science; Tome's Dental Surgery; Harris' Dictionary of Dental Surgery.

### TERMS OF GRADUATION.

A candidate for graduation must have two full years of pupilage, part of which, at least, should be with a reputable dental practitioner, and good teacher, inclusive of two complete courses of lectures in a Dental College.

A graduate of a respectable Medical College who has had one year's pupilage under a reputable dentist, or a student having had a regular pupilage of two years and four years practice, and otherwise satisfactory, may be admitted to the Senior Class, and to examination for the degree of D. D. S., after attending one full course of lectures.

For further particulars address the Dean or Secretary by letter.

Students coming to the city should call on the Dean. Good board can be had for $4.00 to $7.00 a week.

### SPRING COURSE, 1877.

The Spring Course in this Institution will commence on the 15th of March and continue till the 1st of June.

Tickets for the course, $50, to be paid on entering; or $130 for Spring and Winter term. tf

F. BRUNNING, Sec. J. TAFT, Dean, 117 West 4th St. Cin'ti.

### HARVARD UNIVERSITY.
### Dental Department.
### BOSTON, MASS. 1875–76.

FACULTY.

CHARLES WILLIAM ELIOT, L.L.D., *President.*
OLIVER W. HOLMES, M.D., Professor of Anatomy.
HENRY J. BIGELOW, M.D., Professor of Surgery and Clinical Surgery.
THOMAS H. CHANDLER, D.M.D., Professor of Mechanical Dentistry.
GEORGE T. MOFFATT, M.D., D.M.D., Professor of Operative Dentistry.
LUTHER D. SHEPARD, D.D.S., Adjunct Professor of Operative Dentistry.
NATHANIEL W. HAWES, Assistant Professor of Operative Dentistry.
EDWARD S. WOOD, M.D., Assistant Professor of Chemistry.
HENRY P. BOWDITCH, M.D., Assistant Professor of Physiology.
WILLIAM H. ROLLINS, D.M.D., Instructor in Dental Pathology.
CHARLES A. BRACKETT, D.M.D., Instructor in Dental Therapeutics.
IRA A. SALMON, D.D.S., University Lecturer on Operative Dentistry.
CHARLES B. PORTER, M.D., Demonstrator of Practical Anatomy.
CHARLES WILSON, D.M.D., Demonstrator in Charge.
GEORGE F. GRANT, D.M.D., Demonstrator of Mechanical Dentistry.

The Ninth year of this School begins on the 28th of September and continues till the last Wednesday in June. The distinction of Summer and Winter sessions being hereafter abolished. It will be divided into two equal terms, with a recess of one week at Christmas. The course of instruction will be progressive, extending over two years, the lectures of one year not being repeated in the next.

The regular examinations will be held in the following order, viz.: At the end of the first year, anatomy, including dissection, physiology and general chemistry. A certificate from the demonstrator of anatomy will be required of each student, that he has satisfactorily dissected the three parts of the body.

At the end of the second year, dental pathology, including a knowledge of gestation and diseases of women so far as they affect the mouth and throat, dental materia medica and therapeutics, oral surgery and surgical pathology, operative and mechanical dentistry. The examinations in operative and mechanical dentistry will include actual operations and the preparation of specimens of mechanical dentistry.

Every candidate must be twenty-one years of age, and of good moral character; he must give evidence of having studied medicine or dentistry three full years; he must have spent at least one continuous year at this school, have presented a satisfactory thesis and have passed all the required examinations.

The University Degree, D.M.D. (*Dentariæ Medicinæ Doctor*), is conferred upon those who fulfil the requirements.

Students may be admitted to advanced standing upon passing a satisfactory examination in a majority of the studies already pursued by the class; but no student shall advance with his class or be admitted to advance standing until he has passed such examination.

The work in the operative and mechanical infirmaries will go on throughout the course, but no student shall be permitted to operate at the chair until he has by observation and practice on extracted teeth satisfied the Professor of his fitness.

The faculty recommend young men who propose to take the degree to spend the whole of the required term of three years of study in the school. But those who wish to spend but two of the three years in the school are earnestly advised to pass their first year of study, before entering, under the direction of a *competent* private instructor.

Unsurpassed facilities for operative and mechanical practice are furnished by the infirmary of the Massachusetts General Hospital which is open throughout the year.

FEES.

There are no fees for matriculation, for the diploma, nor for the demonstrators. For the first year a student is a member of the school, the fee is $200; for the second year, $150; for any subsequent year, $50.

For further information address,

**THOS. H. CHANDLER, Dean,**

**222 Tremont Street, Boston, Mass.**

### HARVARD UNIVERSITY.

### Dental Department.

### BOSTON, MASS. 1875–76.

---

**FACULTY.**

Charles William Eliot, L.L.D., *President.*
Oliver W. Holmes, M.D., Professor of Anatomy.
Henry J. Bigelow, M.D., Professor of Surgery and Clinical Surgery.
Thomas H. Chandler, D.M.D., Professor of Mechanical Dentistry.
George T. Moffatt, M.D., D.M.D., Professor of Operative Dentistry.
Luther D. Shepard, D.D.S., Adjunct Professor of Operative Dentistry.
Nathaniel W. Hawes, Assistant Professor of Operative Dentistry.
Edward S. Wood, M.D., Assistant Professor of Chemistry.
Henry P. Bowditch, M.D., Assistant Professor of Physiology.
William H. Rollins, D.M.D., Instructor in Dental Pathology.
Charles A. Brackett, D.M.D., Instructor in Dental Therapeutics.
Ira A. Salmon, D.D.S., University Lecturer on Operative Dentistry.
Charles B. Porter, M.D., Demonstrator of Practical Anatomy.
Charles Wilson, D.M.D., Demonstrator in Charge.
George F. Grant, D.M.D., Demonstrator of Mechanical Dentistry.

---

The Ninth year of this School begins on the 28th of September and continues till the last Wednesday in June. The distinction of Summer and Winter sessions being hereafter abolished. It will be divided into two equal terms, with a recess of one week at Christmas. The course of instruction will be progressive, extending over two years, the lectures of one year not being repeated in the next.

The regular examinations will be held in the following order, viz.: At the end of the first year, anatomy, including dissection, physiology and general chemistry. A certificate from the demonstrator of anatomy will be required of each student, that he has satisfactorily dissected the three parts of the body.

At the end of the second year, dental pathology, including a knowledge of gestation and diseases of women so far as they affect the mouth and throat, dental materia medica and therapeutics, oral surgery and surgical pathology, operative and mechanical dentistry. The examinations in operative and mechanical dentistry will include actual operations and the preparation of specimens of mechanical dentistry.

Every candidate must be twenty-one years of age, and of good moral character; he must give evidence of having studied medicine or dentistry three full years; he must have spent at least one continuous year at this school, have presented a satisfactory thesis and have passed all the required examinations.

The University Degree, D.M.D. (*Dentariæ Medicinæ Doctor*), is conferred upon those who fulfil the requirements.

Students may be admitted to advanced standing upon passing a satisfactory examination in a majority of the studies already pursued by the class; but no student shall advance with his class or be admitted to advance standing until he has passed such examination.

The work in the operative and mechanical infirmaries will go on throughout the course, but no student shall be permitted to operate at the chair until he has by observation and practice on extracted teeth satisfied the Professor of his fitness.

The faculty recommend young men who propose to take the degree to spend the whole of the required term of three years of study in the school. But those who wish to spend but two of the three years in the school are earnestly advised to pass their first year of study, before entering, under the direction of a *competent* private instructor.

Unsurpassed facilities for operative and mechanical practice are furnished by the infirmary of the Massachusetts General Hospital which is open throughout the year.

---

**FEES.**

There are no fees for matriculation, for the diploma, nor for the demonstrators. For the first year a student is a member of the school, the fee is $200; for the second year, $150; for any subsequent year, $50.

For further information address,

**THOS. H. CHANDLER, Dean,**

**222 Tremont Street, Boston, Mass.**

### HARVARD UNIVERSITY.

### Dental Department.

### BOSTON, MASS. 1875–76.

### FACULTY.

Charles William Eliot, L.L.D., *President.*
Oliver W. Holmes, M.D., Professor of Anatomy.
Henry J. Bigelow, M.D., Professor of Surgery and Clinical Surgery.
Thomas H. Chandler, D.M.D., Professor of Mechanical Dentistry.
George T. Moffatt, M.D., D.M.D., Professor of Operative Dentistry.
Luther D. Shepard, D.D.S., Adjunct Professor of Operative Dentistry.
Nathaniel W. Hawes, Assistant Professor of Operative Dentistry.
Edward S. Wood, M.D., Assistant Professor of Chemistry.
Henry P. Bowditch, M.D., Assistant Professor of Physiology.
William H. Rollins, D.M.D., Instructor in Dental Pathology.
Charles A. Brackett, D.M.D., Instructor in Dental Therapeutics.
Ira A. Salmon, D.D.S., University Lecturer on Operative Dentistry.
Charles B. Porter, M.D., Demonstrator of Practical Anatomy.
Charles Wilson, D.M.D., Demonstrator in Charge.
George F. Grant, D.M.D., Demonstrator of Mechanical Dentistry.

The Ninth year of this School begins on the 28th of September and continues till the last Wednesday in June. The distinction of Summer and Winter sessions being hereafter abolished. It will be divided into two equal terms, with a recess of one week at Christmas. The course of instruction will be progressive, extending over two years, the lectures of one year not being repeated in the next.

The regular examinations will be held in the following order, viz.: At the end of the first year, anatomy, including dissection, physiology and general chemistry. A certificate from the demonstrator of anatomy will be required of each student, that he has satisfactorily dissected the three parts of the body.

At the end of the second year, dental pathology, including a knowledge of gestation and diseases of women so far as they affect the mouth and throat, dental materia medica and therapeutics, oral surgery and surgical pathology, operative and mechanical dentistry. The examinations in operative and mechanical dentistry will include actual operations and the preparation of specimens of mechanical dentistry.

Every candidate must be twenty-one years of age, and of good moral character; he must give evidence of having studied medicine or dentistry three full years; he must have spent at least one continuous year at this school, have presented a satisfactory thesis and have passed all the required examinations.

The University Degree, D.M.D. (*Dentariæ Medicinæ Doctor*), is conferred upon those who fulfil the requirements.

Students may be admitted to advanced standing upon passing a satisfactory examination in a majority of the studies already pursued by the class; but no student shall advance with his class or be admitted to advance standing until he has passed such examination.

The work in the operative and mechanical infirmaries will go on throughout the course, but no student shall be permitted to operate at the chair until he has by observation and practice on extracted teeth satisfied the Professor of his fitness.

The faculty recommend young men who propose to take the degree to spend the whole of the required term of three years of study in the school. But those who wish to spend but two of the three years in the school are earnestly advised to pass their first year of study, before entering, under the direction of a *competent* private instructor.

Unsurpassed facilities for operative and mechanical practice are furnished by the infirmary of the Massachusetts General Hospital which is open throughout the year.

### FEES.

There are no fees for matriculation, for the diploma, nor for the demonstrators. For the first year a student is a member of the school, the fee is $200; for the second year, $150; for any subsequent year, $50.

For further information address,

**THOS. H. CHANDLER, Dean,**

**222 Tremont Street, Boston, Mass.**

### The Dental College
OF THE
### UNIVERSITY of MICHIGAN

The SECOND ANNUAL SESSION of this Institution will commence on the 1st of October and close on the last Wednesday of March, thus making a course of six months. The regular course of instruction commences at once and will proceed through the term with the customary holiday vacation.

The students in this department will receive instructions in Anatomy, Physiology, Pathology, Chemistry, Materia Medica, Therapeutics and Surgery, from the professors of their respective branches in the *Department of Medicine* and Surgery of the University, when lectures commence and continue the same as with the Dental College.

### FACULTY OF THE DENTAL DEPARTMENT.

J. B. ANGELL, LL. D., President.

J. TAFT, D. D. S., Professor of Principles aud Practice of Operative Dentistry.

J. A. WATLING, D. D, S., Professor of Clinical and Mechanical Dentistry.

W. H. JACKSON, Demonstrator of Mechanical Dentistry.

It is desirable that students should be present at the opening of the session, as the regular course of instruction begins at that time. Seats in the lecture-room are assigned by selection to students in the order of registration on the steward's books; and each student is expected to occupy during the session such seat as he may select. Students on arriving in Ann Arbor should call at the steward's office.

### CONDITION OF GRADUATION.

The candidate must be twenty-one years of age. He must furnish evidence of good moral character.

He must devote three years in the study of his profession, in connection with attendance upon a course of medical lectures. He must attend two full courses of lectures in the Dental College, or one course elsewhere and the last one here, and we recommend that he attend three courses regularly.

He must sustain an examination satisfactory to the Faculty in all the branches taught.

A graduate of the Medical College may enter the senior class, and, if found qualified, may graduate after one year has been devoted to the study of dentistry.

FEES AND EXPENSES.—The fees which must be paid in advance, are as follows:

MATRICULATION FEE.—Residents of Michigan, $10.00; non-residents, $15.00.

ANNUAL DUES.—Residents of Michigan, $15.00; non-residents, $20.00.

GRADUATION FEE.—For all alike $5.00. The admission fee is paid, but once, and entitles the students to the privileges of permanent membership in any department of the University. The annual tax is paid the first year and every year after.

For further particulars address the Dean of the Dental College, Ann Arbor, Michigan.

tf

J. TAFT, Dean.

### OHIO COLLEGE
OF
### DENTAL SURGERY.

The Thirty-First Annual Session of the Ohio College of Dental Surgery will begin on the 18th of October, 1876, and continue till March following.

### FACULTY.

James Taylor, M. D., D. D. S., Emeritus Institutes of Dental Science.
Wm. Clendenin, M. D., Anatomy.
J. L. Cilley, M. D., Physiology and Histology.
F. Brunning, M. D., Pathology and Therapeutics.
J. Taft, D. D. S., Operative Dentistry, Hygiene, Microscopy.
J. S. Cassidy, D. D. S., Chemistry.
H. M. Reid, Clinical Dentistry.
Wm. Van Antwerp, D. D. S., Mechanical Dentistry.
J. L. Cilley, M. D., Practical Anatomy

### SPECIAL PROFESSORS.

Geo. Watt, M. D., D. D. S., Diseases of Women and Children with reference to Dentistry.
F. H. Rehwinkel, M. D., D. D. S., Oral Surgery.

### FEES.

| | |
|---|---|
| Matriculation Fee (but once) | $ 5.00 |
| Professors' Tickets for one session | 100.00 |
| Or for Winter and Spring terms | 130.00 |
| Demonstrator's Ticket (for Anatomy) | 5.00 |
| Diploma Fee | 30.00 |

### TEXT BOOKS.

Gray's or Wilson's Anatomy; Dalton's, Carpenter's or Kirk's Physiology; Wagner's Pathology; U. S. Dispensatory; Fowne's, Turner's or Graham's Chemistry: Watt's Chemical Essays; Taft's Operative Denistry; Richardson's Mechanical Dentistry; Harris' Principles and Practice of Dental Science; Tome's Dental Surgery; Harris' Dictionary of Dental Surgery.

### TERMS OF GRADUATION.

A candidate for graduation must have two full years of pupilage, part of which, at least, should be with a reputable dental practitioner, and good teacher, inclusive of two complete courses of lectures in a Dental College.

A graduate of a respectable Medical College who has had one year's pupilage under a reputable dentist, or a student having had a regular pupilage of two years and four years practice, and otherwise satisfactory, may be admitted to the Senior Class, and to examination for the degree of D. D. S., after attending one full course of lectures.

For further particulars address the Dean or Secretary by letter.

Students coming to the city should call on the Dean. Good board can be had for $4.00 to $7.00 a week.

### SPRING COURSE, 1877.

The Spring Course in this Institution will commence on the 15th of March and continue till the 1st of June.

Tickets for the course, $50, to be paid on entering; or $130 for Spring and Winter term. tf

F. BRUNNING, Sec. J. TAFT, Dean, 117 West 4th St. Cin'ti.

### HARVARD UNIVERSITY.

### Dental Department.

### BOSTON, MASS. 1875–76.

**FACULTY.**

Charles William Eliot, L.L.D., *President.*
Oliver W. Holmes, M.D., Professor of Anatomy.
Henry J. Bigelow, M.D., Professor of Surgery and Clinical Surgery.
Thomas H. Chandler, D.M.D., Professor of Mechanical Dentistry.
George T. Moffatt, M.D., D.M.D., Professor of Operative Dentistry.
Luther D. Shepard, D.D.S., Adjunct Professor of Operative Dentistry.
Nathaniel W. Hawes, Assistant Professor of Operative Dentistry.
Edward S. Wood, M.D., Assistant Professor of Chemistry.
Henry P. Bowditch, M.D., Assistant Professor of Physiology.
William H. Rollins, D.M.D., Instructor in Dental Pathology.
Charles A. Brackett, D.M.D., Instructor in Dental Therapeutics.
Ira A. Salmon, D.D.S., University Lecturer on Operative Dentistry.
Charles B. Porter, M.D., Demonstrator of Practical Anatomy.
Charles Wilson, D.M.D., Demonstrator in Charge.
George F. Grant, D.M.D., Demonstrator of Mechanical Dentistry.

The Ninth year of this School begins on the 28th of September and continues till the last Wednesday in June. The distinction of Summer and Winter sessions being hereafter abolished. It will be divided into two equal terms, with a recess of one week at Christmas. The course of instruction will be progressive, extending over two years, the lectures of one year not being repeated in the next.

The regular examinations will be held in the following order, viz.: At the end of the first year, anatomy, including dissection, physiology and general chemistry. A certificate from the demonstrator of anatomy will be required of each student, that he has satisfactorily dissected the three parts of the body.

At the end of the second year, dental pathology, including a knowledge of gestation and diseases of women so far as they affect the mouth and throat, dental materia medica and therapeutics, oral surgery and surgical pathology, operative and mechanical dentistry. The examinations in operative and mechanical dentistry will include actual operations and the preparation of specimens of mechanical dentistry.

Every candidate must be twenty-one years of age, and of good moral character; he must give evidence of having studied medicine or dentistry three full years; he must have spent at least one continuous year at this school, have presented a satisfactory thesis and have passed all the required examinations.

The University Degree, D.M.D. (*Dentariæ Medicinæ Doctor*), is conferred upon those who fulfil the requirements.

Students may be admitted to advanced standing upon passing a satisfactory examination in a majority of the studies already pursued by the class; but no student shall advance with his class or be admitted to advance standing until he has passed such examination.

The work in the operative and mechanical infirmaries will go on throughout the course; but no student shall be permitted to operate at the chair until he has by observation and practice on extracted teeth satisfied the Professor of his fitness.

The faculty recommend young men who propose to take the degree to spend the whole of the required term of three years of study in the school. But those who wish to spend but two of the three years in the school are earnestly advised to pass their first year of study, before entering, under the direction of a *competent* private instructor.

Unsurpassed facilities for operative and mechanical practice are furnished by the infirmary of the Massachusetts General Hospital which is open throughout the year.

Jan.

**FEES.**

There are no fees for matriculation, for the diploma, nor for the demonstrators. For the first year a student is a member of the school, the fee is $200; for the second year, $150; for any subsequent year, $50.

For further information address,

**THOS. H. CHANDLER, Dean,**

**222 Tremont Street, Boston, Mass.**

### OHIO COLLEGE
OF
### DENTAL SURGERY.

The Thirty-First Annual Session of the Ohio College of Dental Surgery will begin on the 18th of October, 1876, and continue till March following.

### FACULTY.

James Taylor, M. D., D. D. S., Emeritus Institutes of Dental Science.
Wm. Clendenin, M. D., Anatomy.
J. L. Cilley, M. D., Physiology and Histology.
F. Brunning, M. D., Pathology and Therapeutics.
J. Taft, D. D. S., Operative Dentistry, Hygiene, Microscopy.
J. S. Cassidy, D. D. S., Chemistry.
H. M. Reid, Clinical Dentistry.
Wm. Van Antwerp, D. D. S., Mechanical Dentistry.
J. L. Cilley, M. D., Practical Anatomy

### SPECIAL PROFESSORS.

Geo. Watt, M. D., D. D. S., Diseases of Women and Children with reference to Dentistry.
F. H. Rehwinkel, M. D., D. D. S., Oral Surgery.

### FEES.

| | |
|---|---|
| Matriculation Fee (but once)............... | $ 5.00 |
| Professors' Tickets for one session.......... | 100.00 |
| Or for Winter and Spring terms.............. | 130.00 |
| Demonstrator's Ticket (for Anatomy)...... | 5.00 |
| Diploma Fee........................................ | 30.00 |

### TEXT BOOKS.

Gray's or Wilson's Anatomy; Dalton's, Carpenter's or Kirk's Physiology; Wagner's Pathology; U. S. Dispensatory; Fowne's, Turner's or Graham's Chemistry: Watt's Chemical Essays; Taft's Operative Denistry; Richardson's Mechanical Dentistry; Harris' Principles and Practice of Dental Science; Tome's Dental Surgery; Harris' Dictionary of Dental Surgery.

### TERMS OF GRADUATION.

A candidate for graduation must have two full years of pupilage, part of which, at least, should be with a reputable dental practitioner, and good teacher, inclusive of two complete courses of lectures in a Dental College.

A graduate of a respectable Medical College who has had one year's pupilage under a reputable dentist, or a student having had a regular pupilage of two years and four years practice, and otherwise satisfactory, may be admitted to the Senior Class, and to examination for the degree of D. D. S., after attending one full course of lectures.

For further particulars address the Dean or Secretary by letter.

Students coming to the city should call on the Dean. Good board can be had for $4.00 to $7.00 a week.

april

### SPRING COURSE, 1877.

The Spring Course in this Institution will commence on the 15th of March and continue till the 1st of June.

Tickets for the course, $50, to be paid on entering; or $130 for Spring and Winter term. tf

F. BRUNNING, Sec. J. TAFT, Dean, 117 West 4th St. Cin'ti.

### The Dental College

OF THE

### UNIVERSITY of MICHIGAN

The Second Annual Session of this Institution will commence on the 1st of October and close on the last Wednesday of March, thus making a course of six months. The regular course of instruction commences at once and will proceed through the term with the customary holiday vacation.

The students in this department will receive instructions in Anatomy, Physiology, Pathology, Chemistry, Materia Medica, Therapeutics and Surgery, from the professors of their respective branches in the *Department of Medicine* and Surgery of the University, when lectures commence and continue the same as with the Dental College.

### FACULTY OF THE DENTAL DEPARTMENT.

J. B. Angell, LL. D., President.

J. Taft, D. D. S., Professor of Principles aud Practice of Operative Dentistry.

J. A. Watling, D. D, S., Professor of Clinical and Mechanical Dentistry.

W. H. Jackson, Demonstrator of Mechanical Dentistry.

It is desirable that students should be present at the opening of the session, as the regular course of instruction begins at that time. Seats in the lecture-room are assigned by selection to students in the order of registration on the steward's books; and each student is expected to occupy during the session such seat as he may select. Students on arriving in Ann Arbor should call at the steward's office.

### CONDITION OF GRADUATION.

The candidate must be twenty-one years of age. He must furnish evidence of good moral character.

He must devote three years in the study of his profession, in connection with attendance upon a course of medical lectures. He must attend two full courses of lectures in the Dental College, or one course elsewhere and the last one here, and we recommend that he attend three courses regularly.

He must sustain an examination satisfactory to the Faculty in all the branches taught.

A graduate of the Medical College may enter the senior class, and, if found qualified, may graduate after one year has been devoted to the study of dentistry.

Fees and Expenses.—The fees which must be paid in advance, are as follows:

Matriculation Fee.—Residents of Michigan, $10.00; non-residents, $15.00.

Annual Dues.—Residents of Michigan, $15.00; non-residents, $20.00.

Graduation Fee.—For all alike $5.00. The admission fee is paid, but once, and entitles the students to the privileges of permanent membership in any department of the University. The annual tax is paid the first year and every year after.

For further particulars address the Dean of the Dental College, Ann Arbor, Michigan.

tf *april* J. TAFT, Dean.

### HARVARD UNIVERSITY.

### Dental Department.

### BOSTON, MASS. 1875–76.

---

**FACULTY.**

CHARLES WILLIAM ELIOT, L.L.D., *President.*
OLIVER W. HOLMES, M.D., Professor of Anatomy.
HENRY J. BIGELOW, M.D., Professor of Surgery and Clinical Surgery.
THOMAS H. CHANDLER, D.M.D., Professor of Mechanical Dentistry.
GEORGE T. MOFFATT, M.D., D.M.D., Professor of Operative Dentistry.
LUTHER D. SHEPARD, D.D.S., Adjunct Professor of Operative Dentistry.
NATHANIEL W. HAWES, Assistant Professor of Operative Dentistry.
EDWARD S. WOOD, M.D., Assistant Professor of Chemistry.
HENRY P. BOWDITCH, M.D., Assistant Professor of Physiology.
WILLIAM H. ROLLINS, D.M.D., Instructor in Dental Pathology.
CHARLES A. BRACKETT, D.M.D., Instructor in Dental Therapeutics.
IRA A. SALMON, D.D.S., University Lecturer on Operative Dentistry.
CHARLES B. PORTER, M.D., Demonstrator of Practical Anatomy.
CHARLES WILSON, D.M.D., Demonstrator in Charge.
GEORGE F. GRANT, D.M.D., Demonstrator of Mechanical Dentistry.

---

The Ninth year of this School begins on the 28th of September and continues till the last Wednesday in June. The distinction of Summer and Winter sessions being hereafter abolished. It will be divided into two equal terms, with a recess of one week at Christmas. The course of instruction will be progressive, extending over two years, the lectures of one year not being repeated in the next.

The regular examinations will be held in the following order, viz.: At the end of the first year, anatomy, including dissection, physiology and general chemistry. A certificate from the demonstrator of anatomy will be required of each student, that he has satisfactorily dissected the three parts of the body.

At the end of the second year, dental pathology, including a knowledge of gestation and diseases of women so far as they affect the mouth and throat, dental materia medica and therapeutics, oral surgery and surgical pathology, operative and mechanical dentistry. The examinations in operative and mechanical dentistry will include actual operations and the preparation of specimens of mechanical dentistry.

Every candidate must be twenty-one years of age, and of good moral character; he must give evidence of having studied medicine or dentistry three full years; he must have spent at least one continuous year at this school, have presented a satisfactory thesis and have passed all the required examinations.

The University Degree, D.M.D. (*Dentariœ Medicinœ Doctor*), is conferred upon those who fulfil the requirements.

Students may be admitted to advanced standing upon passing a satisfactory examination in a majority of the studies already pursued by the class; but no student shall advance with his class or be admitted to advance standing until he has passed such examination.

The work in the operative and mechanical infirmaries will go on throughout the course; but no student shall be permitted to operate at the chair until he has by observation and practice on extracted teeth satisfied the Professor of his fitness.

The faculty recommend young men who propose to take the degree to spend the whole of the required term of three years of study in the school. But those who wish to spend but two of the three years in the school are earnestly advised to pass their first year of study, before entering, under the direction of a *competent* private instructor.

Unsurpassed facilities for operative and mechanical practice are furnished by the infirmary of the Massachusetts General Hospital which is open throughout the year.

---

Jan.

**FEES.**

There are no fees for matriculation, for the diploma, nor for the demonstrators. For the first year a student is a member of the school, the fee is $200; for the second year, $150; for any subsequent year, $50.

For further information address,

**THOS. H. CHANDLER, Dean,**

**222 Tremont Street, Boston, Mass.**

### OHIO COLLEGE

OF

### DENTAL SURGERY.

The THIRTY-FIRST ANNUAL SESSION of the OHIO COLLEGE OF DENTAL SURGERY will begin on the 18th of October, 1876, and continue till March following.

### FACULTY.

JAMES TAYLOR, M. D., D. D. S., Emeritus Institutes of Dental Science.
WM. CLENDENIN, M. D., Anatomy.
J. L, CILLEY, M. D., Physiology and Histology.
F. BRUNNING, M. D., Pathology and Therapeutics.
J. TAFT, D. D. S., Operative Dentistry, Hygiene, Microscopy.
J. S. CASSIDY, D. D. S., Chemistry.
H. M. REID, Clinical Dentistry.
WM. VAN ANTWERP, D. D. S., Mechanical Dentistry.
J. L. CILLEY, M. D., Practical Anatomy

### SPECIAL PROFESSORS.

GEO. WATT, M. D., D. D. S., Diseases of Women and Children with reference to Dentistry.
F. H. REHWINKEL, M. D., D. D. S., Oral Surgery.

### FEES.

| | |
|---|---|
| Matriculation Fee (but once)............... | $ 5.00 |
| Professors' Tickets for one session.......... | 100.00 |
| Or for Winter and Spring terms............. | 130.00 |
| Demonstrator's Ticket (for Anatomy)...... | 5.00 |
| Diploma Fee.......................................... | 30.00 |

### TEXT BOOKS.

Gray's or Wilson's Anatomy; Dalton's, Carpenter's or Kirk's Physiology; Wagner's Pathology; U. S. Dispensatory; Fowne's, Turner's or Graham's Chemistry: Watt's Chemical Essays; Taft's Operative Denistry; Richardson's Mechanical Dentistry; Harris' Principles and Practice of Dental Science; Tome's Dental Surgery; Harris' Dictionary of Dental Surgery.

### TERMS OF GRADUATION.

A candidate for graduation must have two full years of pupilage, part of which, at least, should be with a reputable dental practitioner, and good teacher, inclusive of two complete courses of lectures in a Dental College.

A graduate of a respectable Medical College who has had one year's pupilage under a reputable dentist, or a student having had a regular pupilage of two years and four years practice, and otherwise satisfactory, may be admitted to the Senior Class, and to examination for the degree of D. D. S., after attending one full course of lectures.

For further particulars address the Dean or Secretary by letter.

Students coming to the city should call on the Dean. Good board can be had for $4.00 to $7.00 a week.

april

### SPRING COURSE, 1877.

The Spring Course in this Institution will commence on the 15th of March and continue till the 1st of June.

Tickets for the course, $50, to be paid on entering; or $130 for Spring and Winter term. tf

F. BRUNNING, SEC. J. TAFT, DEAN, 117 West 4th St. Cin'ti.

### The Dental College

OF THE

### UNIVERSITY of MICHIGAN

The SECOND ANNUAL SESSION of this Institution will commence on the 1st of October and close on the last Wednesday of March, thus making a course of six months. The regular course of instruction commences at once and will proceed through the term with the customary holiday vacation.

The students in this department will receive instructions in Anatomy, Physiology, Pathology, Chemistry, Materia Medica, Therapeutics and Surgery, from the professors of their respective branches in the *Department of Medicine* and Surgery of the University, when lectures commence and continue the same as with the Dental College.

### FACULTY OF THE DENTAL DEPARTMENT.

J. B. ANGELL, LL. D., President.

J. TAFT, D. D. S., Professor of Principles aud Practice of Operative Dentistry.

J. A. WATLING, D. D, S., Professor of Clinical and Mechanical Dentistry.

W. H. JACKSON, Demonstrator of Mechanical Dentistry.

It is desirable that students should be present at the opening of the session, as the regular course of instruction begins at that time. Seats in the lecture-room are assigned by selection to students in the order of registration on the steward's books; and each student is expected to occupy during the session such seat as he may select. Students on arriving in Ann Arbor should call at the steward's office.

### CONDITION OF GRADUATION.

The candidate must be twenty-one years of age. He must furnish evidence of good moral character.

He must devote three years in the study of his profession, in connection with attendance upon a course of medical lectures. He must attend two full courses of lectures in the Dental College, or one course elsewhere and the last one here, and we recommend that he attend three courses regularly.

He must sustain an examination satisfactory to the Faculty in all the branches taught.

A graduate of the Medical College may enter the senior class, and, if found qualified, may graduate after one year has been devoted to the study of dentistry.

FEES AND EXPENSES.—The fees which must be paid in advance, are as follows:

MATRICULATION FEE.—Residents of Michigan, $10.00; non-residents, $15.00.

ANNUAL DUES.—Residents of Michigan, $15.00; non-residents, $20.00.

GRADUATION FEE.—For all alike $5.00. The admission fee is paid, but once, and entitles the students to the privileges of permanent membership in any department of the University. The annual tax is paid the first year and every year after.

For further particulars address the Dean of the Dental College, Ann Arbor, Michigan.

tf apri' J. TAFT, Dean.

### The Dental College

OF THE

### UNIVERSITY of MICHIGAN

The Second Annual Session of this Institution will commence on the 1st of October and close on the last Wednesday of March, thus making a course of six months. The regular course of instruction commences at once and will proceed through the term with the customary holiday vacation.

The students in this department will receive instructions in Anatomy, Physiology, Pathology, Chemistry, Materia Medica, Therapeutics and Surgery, from the professors of their respective branches in the *Department of Medicine* and Surgery of the University, when lectures commence and continue the same as with the Dental College.

### FACULTY OF THE DENTAL DEPARTMENT.

J. B. Angell, LL. D., President.

J. Taft, D. D. S., Professor of Principles aud Practice of Operative Dentistry.

J. A. Watling, D. D, S., Professor of Clinical and Mechanical Dentistry.

W. H. Jackson, Demonstrator of Mechanical Dentistry.

It is desirable that students should be present at the opening of the session, as the regular course of instruction begins at that time. Seats in the lecture-room are assigned by selection to students in the order of registration on the steward's books; and each student is expected to occupy during the session such seat as he may select. Students on arriving in Ann Arbor should call at the steward's office.

### CONDITION OF GRADUATION.

The candidate must be twenty-one years of age. He must furnish evidence of good moral character.

He must devote three years in the study of his profession, in connection with attendance upon a course of medical lectures. He must attend two full courses of lectures in the Dental College, or one course elsewhere and the last one here, and we recommend that he attend three courses regularly.

He must sustain an examination satisfactory to the Faculty in all the branches taught.

A graduate of the Medical College may enter the senior class, and, if found qualified, may graduate after one year has been devoted to the study of dentistry.

Fees and Expenses.—The fees which must be paid in advance, are as follows:

Matriculation Fee.—Residents of Michigan, $10.00; non-residents, $15.00.

Annual Dues.—Residents of Michigan, $15.00; non-residents, $20.00.

Graduation Fee.—For all alike $5.00. The admission fee is paid, but once, and entitles the students to the privileges of permanent membership in any department of the University. The annual tax is paid the first year and every year after.

For further particulars address the Dean of the Dental College, Ann Arbor, Michigan.

tf J. TAFT, Dean.

Sept-5

### HARVARD UNIVERSITY.

### Dental Department.

### BOSTON, MASS. 1875–76.

---

**FACULTY.**

Charles William Eliot, L.L.D., *President.*
Oliver W. Holmes, M.D., Professor of Anatomy.
Henry J. Bigelow, M.D., Professor of Surgery and Clinical Surgery.
Thomas H. Chandler, D.M.D., Professor of Mechanical Dentistry.
George T. Moffatt, M.D., D.M.D., Professor of Operative Dentistry.
Luther D. Shepard, D.D.S., Adjunct Professor of Operative Dentistry.
Nathaniel W. Hawes, Assistant Professor of Operative Dentistry.
Edward S. Wood, M.D., Assistant Professor of Chemistry.
Henry P. Bowditch, M.D., Assistant Professor of Physiology.
William H. Rollins, D.M.D., Instructor in Dental Pathology.
Charles A. Brackett, D.M.D., Instructor in Dental Therapeutics.
Ira A. Salmon, D.D.S., University Lecturer on Operative Dentistry.
Charles B. Porter, M.D., Demonstrator of Practical Anatomy.
Charles Wilson, D.M.D., Demonstrator in Charge.
George F. Grant, D.M.D., Demonstrator of Mechanical Dentistry.

---

The Ninth year of this School begins on the 28th of September and continues till the last Wednesday in June. The distinction of Summer and Winter sessions being hereafter abolished. It will be divided into two equal terms, with a recess of one week at Christmas. The course of instruction will be progressive, extending over two years, the lectures of one year not being repeated in the next.

The regular examinations will be held in the following order, viz.: At the end of the first year, anatomy, including dissection, physiology and general chemistry. A certificate from the demonstrator of anatomy will be required of each student, that he has satisfactorily dissected the three parts of the body.

At the end of the second year, dental pathology, including a knowledge of gestation and diseases of women so far as they affect the mouth and throat, dental materia medica and therapeutics, oral surgery and surgical pathology, operative and mechanical dentistry. The examinations in operative and mechanical dentistry will include actual operations and the preparation of specimens of mechanical dentistry.

Every candidate must be twenty-one years of age, and of good moral character; he must give evidence of having studied medicine or dentistry three full years; he must have spent at least one continuous year at this school, have presented a satisfactory thesis and have passed all the required examinations.

The University Degree, D.M.D. (*Dentariæ Medicinæ Doctor*), is conferred upon those who fulfil the requirements.

Students may be admitted to advanced standing upon passing a satisfactory examination in a majority of the studies already pursued by the class; but no student shall advance with his class or be admitted to advance standing until he has passed such examination.

The work in the operative and mechanical infirmaries will go on throughout the course; but no student shall be permitted to operate at the chair until he has by observation and practice on extracted teeth satisfied the Professor of his fitness.

The faculty recommend young men who propose to take the degree to spend the whole of the required term of three years of study in the school. But those who wish to spend but two of the three years in the school are earnestly advised to pass their first year of study, before entering, under the direction of a *competent* private instructor.

Unsurpassed facilities for operative and mechanical practice are furnished by the infirmary of the Massachusetts General Hospital which is open throughout the year.

---

Jan.

**FEES.**

There are no fees for matriculation, for the diploma, nor for the demonstrators. For the first year a student is a member of the school, the fee is $200; for the second year, $150; for any subsequent year, $50.

For further information address,

**THOS. H. CHANDLER, Dean,**

**222 Tremont Street, Boston, Mass.**

### OHIO COLLEGE
OF
### DENTAL SURGERY.

The Thirty-First Annual Session of the Ohio College of Dental Surgery will begin on the 18th of October, 1876, and continue till March following.

### FACULTY.

James Taylor, M. D., D. D. S., Emeritus Institutes of Dental Science.
Wm. Clendenin, M. D., Anatomy.
J. L. Cilley, M. D., Physiology and Histology.
F. Brunning, M. D., Pathology and Therapeutics.
J. Taft, D. D. S., Operative Dentistry, Hygiene, Microscopy.
J. S. Cassidy, D. D. S., Chemistry.
H. M. Reid, Clinical Dentistry.
Wm. Van Antwerp, D. D. S., Mechanical Dentistry.
J. L. Cilley, M. D., Practical Anatomy.

### SPECIAL PROFESSORS.

Geo. Watt, M. D., D. D. S., Diseases of Women and Children with reference to Dentistry.
F. H. Rehwinkel, M. D., D. D. S., Oral Surgery.

### FEES.

| | |
|---|---|
| Matriculation Fee (but once)............... | $ 5.00 |
| Professors' Tickets for one session.......... | 100.00 |
| Or for Winter and Spring terms............... | 130.00 |
| Demonstrator's Ticket (for Anatomy)...... | 5.00 |
| Diploma Fee........................................ | 30.00 |

### TEXT BOOKS.

Gray's or Wilson's Anatomy; Dalton's, Carpenter's or Kirk's Physiology; Wagner's Pathology; U. S. Dispensatory; Fowne's, Turner's or Graham's Chemistry: Watt's Chemical Essays; Taft's Operative Denistry; Richardson's Mechanical Dentistry; Harris' Principles and Practice of Dental Science; Tome's Dental Surgery; Harris' Dictionary of Dental Surgery.

### TERMS OF GRADUATION.

A candidate for graduation must have two full years of pupilage, part of which, at least, should be with a reputable dental practitioner, and good teacher, inclusive of two complete courses of lectures in a Dental College.

A graduate of a respectable Medical College who has had one year's pupilage under a reputable dentist, or a student having had a regular pupilage of two years and four years practice, and otherwise satisfactory, may be admitted to the Senior Class, and to examination for the degree of D. D. S., after attending one full course of lectures.

For further particulars address the Dean or Secretary by letter.

Students coming to the city should call on the Dean. Good board can be had for $4.00 to $7.00 a week.

april

### SPRING COURSE, 1877.

The Spring Course in this Institution will commence on the 15th of March and continue till the 1st of June.

Tickets for the course, $50, to be paid on entering; or $130 for Spring and Winter term. tf

F. BRUNNING, Sec. J. TAFT, Dean, 117 West 4th St. Cin'ti.

### The Dental College

OF THE

### UNIVERSITY of MICHIGAN

The SECOND ANNUAL SESSION of this Institution will commence on the 1st of October and close on the last Wednesday of March, thus making a course of six months. The regular course of instruction commences at once and will proceed through the term with the customary holiday vacation.

The students in this department will receive instructions in Anatomy, Physiology, Pathology, Chemistry, Materia Medica, Therapeutics and Surgery, from the professors of their respective branches in the *Department of Medicine* and Surgery of the University, when lectures commence and continue the same as with the Dental College.

### FACULTY OF THE DENTAL DEPARTMENT.

J. B. ANGELL, LL. D., President.

J. TAFT, D. D. S., Professor of Principles aud Practice of Operative Dentistry.

J. A. WATLING, D. D, S., Professor of Clinical and Mechanical Dentistry.

W. H. JACKSON, Demonstrator of Mechanical Dentistry.

It is desirable that students should be present at the opening of the session, as the regular course of instruction begins at that time. Seats in the lecture-room are assigned by selection to students in the order of registration on the steward's books; and each student is expected to occupy during the session such seat as he may select. Students on arriving in Ann Arbor should call at the steward's office.

### CONDITION OF GRADUATION.

The candidate must be twenty-one years of age. He must furnish evidence of good moral character.

He must devote three years in the study of his profession, in connection with attendance upon a course of medical lectures. He must attend two full courses of lectures in the Dental College, or one course elsewhere and the last one here, and we recommend that he attend three courses regularly.

He must sustain an examination satisfactory to the Faculty in all the branches taught.

A graduate of the Medical College may enter the senior class, and, if found qualified, may graduate after one year has been devoted to the study of dentistry.

FEES AND EXPENSES.—The fees which must be paid in advance, are as follows:

MATRICULATION FEE.—Residents of Michigan, $10.00; non-residents, $15.00.

ANNUAL DUES.—Residents of Michigan, $15.00; non-residents, $20.00.

GRADUATION FEE.—For all alike $5.00. The admission fee is paid, but once, and entitles the students to the privileges of permanent membership in any department of the University. The annual tax is paid the first year and every year after.

For further particulars address the Dean of the Dental College, Ann Arbor, Michigan.

tf — J. TAFT, Dean.

Oct-5

### HARVARD UNIVERSITY.

### Dental Department.

### BOSTON, MASS. 1875–76.

---

**FACULTY.**

Charles William Eliot, L.L.D., *President.*
Oliver W. Holmes, M.D., Professor of Anatomy.
Henry J. Bigelow, M.D., Professor of Surgery and Clinical Surgery.
Thomas H. Chandler, D.M.D., Professor of Mechanical Dentistry.
George T. Moffatt, M.D., D.M.D., Professor of Operative Dentistry.
Luther D. Shepard, D.D.S., Adjunct Professor of Operative Dentistry.
Nathaniel W. Hawes, Assistant Professor of Operative Dentistry.
Edward S. Wood, M.D., Assistant Professor of Chemistry.
Henry P. Bowditch, M.D., Assistant Professor of Physiology.
William H. Rollins, D.M.D., Instructor in Dental Pathology.
Charles A. Brackett, D.M.D., Instructor in Dental Therapeutics.
Ira A. Salmon, D.D.S., University Lecturer on Operative Dentistry.
Charles B. Porter, M.D., Demonstrator of Practical Anatomy.
Charles Wilson, D.M.D., Demonstrator in Charge.
George F. Grant, D.M.D., Demonstrator of Mechanical Dentistry.

---

The Ninth year of this School begins on the 28th of September and continues till the last Wednesday in June. The distinction of Summer and Winter sessions being hereafter abolished. It will be divided into two equal terms, with a recess of one week at Christmas. The course of instruction will be progressive, extending over two years, the lectures of one year not being repeated in the next.

The regular examinations will be held in the following order, viz.: At the end of the first year, anatomy, including dissection, physiology and general chemistry. A certificate from the demonstrator of anatomy will be required of each student, that he has satisfactorily dissected the three parts of the body.

At the end of the second year, dental pathology, including a knowledge of gestation and diseases of women so far as they affect the mouth and throat, dental materia medica and therapeutics, oral surgery and surgical pathology, operative and mechanical dentistry. The examinations in operative and mechanical dentistry will include actual operations and the preparation of specimens of mechanical dentistry.

Every candidate must be twenty-one years of age, and of good moral character; he must give evidence of having studied medicine or dentistry three full years; he must have spent at least one continuous year at this school, have presented a satisfactory thesis and have passed all the required examinations.

The University Degree, D.M.D. (*Dentariæ Medicinæ Doctor*), is conferred upon those who fulfil the requirements.

Students may be admitted to advanced standing upon passing a satisfactory examination in a majority of the studies already pursued by the class; but no student shall advance with his class or be admitted to advance standing until he has passed such examination.

The work in the operative and mechanical infirmaries will go on throughout the course, but no student shall be permitted to operate at the chair until he has by observation and practice on extracted teeth satisfied the Professor of his fitness.

The faculty recommend young men who propose to take the degree to spend the whole of the required term of three years of study in the school. But those who wish to spend but two of the three years in the school are earnestly advised to pass their first year of study, before entering, under the direction of a *competent* private instructor.

Unsurpassed facilities for operative and mechanical practice are furnished by the infirmary of the Massachusetts General Hospital which is open throughout the year.

---

Jan.

**FEES.**

There are no fees for matriculation, for the diploma, nor for the demonstrators. For the first year a student is a member of the school, the fee is $200; for the second year, $150; for any subsequent year, $50.

For further information address,

**THOS. H. CHANDLER, Dean,**

**222 Tremont Street, Boston, Mass.**

### OHIO COLLEGE

OF

### DENTAL SURGERY.

The THIRTY-FIRST ANNUAL SESSION of the OHIO COLLEGE OF DENTAL SURGERY will begin on the 18th of October, 1876, and continue till March following.

### FACULTY.

JAMES TAYLOR, M. D., D. D. S., Emeritus Institutes of Dental Science.
WM. CLENDENIN, M. D., Anatomy.
J. L. CILLEY, M. D., Physiology and Histology.
F. BRUNNING, M. D., Pathology and Therapeutics.
J. TAFT, D. D. S., Operative Dentistry, Hygiene, Microscopy.
J. S. CASSIDY, D. D. S., Chemistry.
H. M. REID, Clinical Dentistry.
WM. VAN ANTWERP, D. D. S., Mechanical Dentistry.
J. L. CILLEY, M. D., Practical Anatomy

### SPECIAL PROFESSORS.

GEO. WATT, M. D., D. D. S., Diseases of Women and Children with reference to Dentistry.
F. H. REHWINKEL, M. D., D. D. S., Oral Surgery.

### FEES.

| | |
|---|---|
| Matriculation Fee (but once)............... | $ 5.00 |
| Professors' Tickets for one session.......... | 100.00 |
| Or for Winter and Spring terms.............. | 130.00 |
| Demonstrator's Ticket (for Anatomy)...... | 5.00 |
| Diploma Fee............................................ | 30.00 |

### TEXT BOOKS.

Gray's or Wilson's Anatomy; Dalton's, Carpenter's or Kirk's Physiology; Wagner's Pathology; U. S. Dispensatory; Fowne's, Turner's or Graham's Chemistry: Watt's Chemical Essays; Taft's Operative Denistry; Richardson's Mechanical Dentistry; Harris' Principles and Practice of Dental Science; Tome's Dental Surgery; Harris' Dictionary of Dental Surgery.

### TERMS OF GRADUATION.

A candidate for graduation must have two full years of pupilage, part of which, at least, should be with a reputable dental practitioner, and good teacher, inclusive of two complete courses of lectures in a Dental College.

A graduate of a respectable Medical College who has had one year's pupilage under a reputable dentist, or a student having had a regular pupilage of two years and four years practice, and otherwise satisfactory, may be admitted to the Senior Class, and to examination for the degree of D. D. S., after attending one full course of lectures.

For further particulars address the Dean or Secretary by letter.

Students coming to the city should call on the Dean. Good board can be had for $4.00 to $7.00 a week.

pril

### SPRING COURSE, 1877.

The Spring Course in this Institution will commence on the 15th of March and continue till the 1st of June.

Tickets for the course, $50, to be paid on entering; or $130 for Spring and Winter term. tf

F. BRUNNING, SEC. J. TAFT, DEAN, 117 West 4th St. Cin'ti.

### The Dental College

OF THE

### UNIVERSITY of MICHIGAN

The SECOND ANNUAL SESSION of this Institution will commence on the 1st of October and close on the last Wednesday of March, thus making a course of six months. The regular course of instruction commences at once and will proceed through the term with the customary holiday vacation.

The students in this department will receive instructions in Anatomy, Physiology, Pathology, Chemistry, Materia Medica, Therapeutics and Surgery, from the professors of their respective branches in the *Department of Medicine* and Surgery of the University, when lectures commence and continue the same as with the Dental College.

### FACULTY OF THE DENTAL DEPARTMENT.

J. B. ANGELL, LL. D., President.

J. TAFT, D. D. S., Professor of Principles aud Practice of Operative Dentistry.

J. A. WATLING, D. D, S., Professor of Clinical and Mechanical Dentistry.

W. H. JACKSON, Demonstrator of Mechanical Dentistry.

It is desirable that students should be present at the opening of the session, as the regular course of instruction begins at that time. Seats in the lecture-room are assigned by selection to students in the order of registration on the steward's books; and each student is expected to occupy during the session such seat as he may select. Students on arriving in Ann Arbor should call at the steward's office.

### CONDITION OF GRADUATION.

The candidate must be twenty-one years of age. He must furnish evidence of good moral character.

He must devote three years in the study of his profession, in connection with attendance upon a course of medical lectures. He must attend two full courses of lectures in the Dental College, or one course elsewhere and the last one here, and we recommend that he attend three courses regularly.

He must sustain an examination satisfactory to the Faculty in all the branches taught.

A graduate of the Medical College may enter the senior class, and, if found qualified, may graduate after one year has been devoted to the study of dentistry.

FEES AND EXPENSES.—The fees which must be paid in advance, are as follows:

MATRICULATION FEE.—Residents of Michigan, $10.00; non-residents, $15.00.

ANNUAL DUES.—Residents of Michigan, $15.00; non-residents, $20.00.

GRADUATION FEE.—For all alike $5.00. The admission fee is paid, but once, and entitles the students to the privileges of permanent membership in any department of the University. The annual tax is paid the first year and every year after.

For further particulars address the Dean of the Dental College, Ann Arbor, Michigan.

tf J. TAFT, Dean.

### HARVARD UNIVERSITY.

### Dental Department.

### BOSTON, MASS. 1875–76.

### FACULTY.

CHARLES WILLIAM ELIOT, L.L.D., *President.*
OLIVER W. HOLMES, M.D., Professor of Anatomy.
HENRY J. BIGELOW, M.D., Professor of Surgery and Clinical Surgery.
THOMAS H. CHANDLER, D.M.D., Professor of Mechanical Dentistry.
GEORGE T. MOFFATT, M.D., D.M.D., Professor of Operative Dentistry.
LUTHER D. SHEPARD, D.D.S., Adjunct Professor of Operative Dentistry.
NATHANIEL W. HAWES, Assistant Professor of Operative Dentistry.
EDWARD S. WOOD, M.D., Assistant Professor of Chemistry.
HENRY P. BOWDITCH, M.D., Assistant Professor of Physiology.
WILLIAM H. ROLLINS, D.M.D., Instructor in Dental Pathology.
CHARLES A. BRACKETT, D.M.D., Instructor in Dental Therapeutics.
IRA A. SALMON, D.D.S., University Lecturer on Operative Dentistry.
CHARLES B. PORTER, M.D., Demonstrator of Practical Anatomy.
CHARLES WILSON, D.M.D., Demonstrator in Charge.
GEORGE F. GRANT, D.M.D., Demonstrator of Mechanical Dentistry.

---

The Ninth year of this School begins on the 28th of September and continues till the last Wednesday in June. The distinction of Summer and Winter sessions being hereafter abolished. It will be divided into two equal terms, with a recess of one week at Christmas. The course of instruction will be progressive, extending over two years, the lectures of one year not being repeated in the next.

The regular examinations will be held in the following order, viz.: At the end of the first year, anatomy, including dissection, physiology and general chemistry. A certificate from the demonstrator of anatomy will be required of each student, that he has satisfactorily dissected the three parts of the body.

At the end of the second year, dental pathology, including a knowledge of gestation and diseases of women so far as they affect the mouth and throat, dental materia medica and therapeutics, oral surgery and surgical pathology, operative and mechanical dentistry. The examinations in operative and mechanical dentistry will include actual operations and the preparation of specimens of mechanical dentistry.

Every candidate must be twenty-one years of age, and of good moral character; he must give evidence of having studied medicine or dentistry three full years; he must have spent at least one continuous year at this school, have presented a satisfactory thesis and have passed all the required examinations.

The University Degree, D.M.D. (*Dentariæ Medicinæ Doctor*), is conferred upon those who fulfil the requirements.

Students may be admitted to advanced standing upon passing a satisfactory examination in a majority of the studies already pursued by the class; but no student shall advance with his class or be admitted to advance standing until he has passed such examination.

The work in the operative and mechanical infirmaries will go on throughout the course, but no student shall be permitted to operate at the chair until he has by observation and practice on extracted teeth satisfied the Professor of his fitness.

The faculty recommend young men who propose to take the degree to spend the whole of the required term of three years of study in the school. But those who wish to spend but two of the three years in the school are earnestly advised to pass their first year of study, before entering, under the direction of a *competent* private instructor.

Unsurpassed facilities for operative and mechanical practice are furnished by the infirmary of the Massachusetts General Hospital which is open throughout the year.

---

Jan.

### FEES.

There are no fees for matriculation, for the diploma, nor for the demonstrators. For the first year a student is a member of the school, the fee is $200; for the second year, $150; for any subsequent year, $50.

For further information address,

**THOS. H. CHANDLER, Dean,**

**222 Tremont Street, Boston, Mass.**

### OHIO COLLEGE
OF
### DENTAL SURGERY.

The THIRTY-FIRST ANNUAL SESSION of the OHIO COLLEGE OF DENTAL SURGERY will begin on the 18th of October, 1876, and continue till March following.

### FACULTY.

JAMES TAYLOR, M. D., D. D. S., Emeritus Institutes of Dental Science.
WM. CLENDENIN, M. D., Anatomy.
J. L. CILLEY, M. D., Physiology and Histology.
F. BRUNNING, M. D., Pathology and Therapeutics.
J. TAFT, D. D. S., Operative Dentistry, Hygiene, Microscopy.
J. S. CASSIDY, D. D. S., Chemistry.
H. M. REID, Clinical Dentistry.
WM. VAN ANTWERP, D. D. S., Mechanical Dentistry.
J. L. CILLEY, M. D., Practical Anatomy

### SPECIAL PROFESSORS.

GEO. WATT, M. D., D. D. S., Diseases of Women and Children with reference to Dentistry.
F. H. REHWINKEL, M. D., D. D. S., Oral Surgery.

### FEES.

| | |
|---|---|
| Matriculation Fee (but once)............... | $ 5.00 |
| Professors' Tickets for one session.......... | 100.00 |
| Or for Winter and Spring terms.............. | 130.00 |
| Demonstrator's Ticket (for Anatomy)...... | 5.00 |
| Diploma Fee........................................ | 30.00 |

### TEXT BOOKS.

Gray's or Wilson's Anatomy; Dalton's, Carpenter's or Kirk's Physiology; Wagner's Pathology; U. S. Dispensatory; Fowne's, Turner's or Graham's Chemistry: Watt's Chemical Essays; Taft's Operative Dentistry; Richardson's Mechanical Dentistry; Harris' Principles and Practice of Dental Science; Tome's Dental Surgery; Harris' Dictionary of Dental Surgery.

### TERMS OF GRADUATION.

A candidate for graduation must have two full years of pupilage, part of which, at least, should be with a reputable dental practitioner, and good teacher, inclusive of two complete courses of lectures in a Dental College.

A graduate of a respectable Medical College who has had one year's pupilage under a reputable dentist, or a student having had a regular pupilage of two years and four years practice, and otherwise satisfactory, may be admitted to the Senior Class, and to examination for the degree of D. D. S., after attending one full course of lectures.

For further particulars address the Dean or Secretary by letter.

Students coming to the city should call on the Dean. Good board can be had for $4.00 to $7.00 a week.

pril

### SPRING COURSE, 1877.

The Spring Course in this Institution will commence on the 15th of March and continue till the 1st of June.

Tickets for the course, $50, to be paid on entering; or $130 for Spring and Winter term. tf

F. BRUNNING, SEC. J. TAFT, DEAN, 117 West 4th St. Cin'ti.

AMERICAN DENTAL ASSOCIATION—Meets on the first Tuesday in Aug., 1877, at Chicago. *Pres.* G. W. Keeley, Oxford, Ohio; *Cor. Sec.* J. H. McQuilien, Philadel'a; *Rec. Sec.* C. Stoddard Smith, Springfield, Ill.

*American Dental Convention* Meets at Deer Park, Md., second Tuesday in August, 1877. *Pres.* W H. Atkinson, New York; *Cor. Sec.* G. W. Mills, Baltimore; *Rec. Sec.* A. Tees, Philadelphia, Pa.

*List of Societies Represented in the American Dental Association.*

AMERICAN ACADEMY OF DENTAL SURGERY met in New York April 7, 1875. *Pres.* Geo. H. Perine; *Rec. Sec.* N. M. Beckwith; *Cor. Sec.* J. A. Bishop.

*American Academy of Dental Science* Meets at Boston, Mass. *Pres.* Dr. D. M. Parker. *Sec.* W. L. Tucker, of Boston; *Cor. Sec.*, Geo. T. Moffatt.

*Baltimore Dental Society—Pres.* C. T. Brockett; *Rec. Sec.* F. F. Drew.

*Brooklyn Dental Society* Meets semi-monthly. *Pres.* H. G. Mesick. *Rec. Sec* C. P. Crandell; *Cor. Sec.* Wm. Fishbough.

*Buffalo Dental Society*Meets second Monday of each month at Buffalo. *Pres.* S. A. FREEMAN; *Rec. Sec.* G. C. DABOLL.

*Boston Dental Institute*—Meets first Tuesday of each month. *Pres.* D. G. WILLIAMS, Buffalo; *Cor. Sec.* D. S. Dickerman.

*California State Dental Association* Meets annually in June. *Pres.* C. C. Knowles, San Francisco; *Rec. Sec.* H. J. Plomteaux, Woodland.

*Chicago Dental Society* Meets monthly at Chicago. *Pres.* C. R. E. Koch; *Rec. Sec.* D. B. Freeman.

*Connecticut Valley Dental Association.* Meets semi-annually, second Tuesday of June and October. *Pres.* H. F. Bishop, Worcester, Mass.; *Rec. Sec.* C. T. Stockwell, Westfield, Mass. Next Meeting in Springfield, Mass., Oct. 17, 1876.

*Connecticut State Dental Society. Pres.* R. C. Dunham, New Britain; *Rec. Sec.*, D. E. Lane, Hartford.

*Charleston Dental Association*—Meets second Tuesday of every month; *Pres.* T. F. Chupein; *Sec. and Treas.* B. A. Muckenfuss.

*Central Penn. Dental Association* Meets at Huntingdon, Jan., 1876. *Pres.* E. J. Green; *Sec.* W. B. Miller.

CINCINNATI DENTAL SOCIETY—Meets first and third Tuesday of each month. *Pres.* A. Berry; *Sec.* A. Downing.

*Delaware Dental Association*- -Meets semi-annually. *Pres.* E. W. Haines, Newark; *Sec.* C. R. Jeffries, Wilmington.

*Dental Society of the State of Maryland and District of Columbia* Meets on Wednesday, Sept. 6, 1876, in Baltimore. *Pres.* R. B. Winder; *Rec. Sec.* R. F. Hunt; *Cor. Sec.* E. P. Keech.

*Eastern Indiana Dental Association.*—Meets semi-annually, first Tuesday in May and last Tuesday in October; *Pres.* J. K. Jameson, Connersville; *Sec.* M. H. Chappee, Knightstown, Ind. (Place of meeting, Connersville.

*Central Ohio Dental Society* Meets at Crestline, on the 2d Monday in April, 1876. *Pres.* M. DeCamp; *Sec.* N. J. Cressinger.

*East Tennessee Dental Association*—Meets at Chattanooga, 3d Wednesday of Sept., 1876. *Pres.* S. M. Prothro; *Rec. Sec.*, S. B. Cooke.

*Georgia State Dental Society* Next meeting at Atlanta, 2d Tuesday in May, 1877. *Pres.* L. D. Carpenter. *Cor. Sec.* Dr. M. S. Jobson.

*Harris Dental Association* Meets quarterly on the first Thursday in February, May, August, Nov'ber. *Pres.* E. K. Young; *Sec.* W. N. Amer, Lancaster.

*Hudson Valley Dental Association* Meets monthly at Troy, N. Y. *Pres.* L. C. Wheeler, *Rec. Sec.* S. G. Andres; *Cor. Sec.* S. D. French.

*Illinois State Dental Soeiety* Meets annually. *Pres.* K. B. Davis, Springfield; *Sec.* E. D. Swain, Chicago. Meets second Tuesday in May, 1877, at Springfield.

*Indiana State Dental Society*—Meets next year at Ft. Wayne, on the last Tuesday of June, 1877. *Pres.* Isaac Knapp, Ft. Wayne; *Sec.* J. E. Cravens.

*Iowa State Dental Society*- -Meets annually. Next meeting at Burlington, on the second Tuesday of May. *Pres.* C. P. Artman Waterloo; *Rec. Sec.* R. L. Cochran Burlington; *Cor. Sec.* I. P. Wilson, Burlington.

*Kansas State Dental Society* Meets annually. Next meeting at Leavanworth, first Wednesday in May, 1877. *Pres.* J. D Patterson, Lawrence; *Sec.* W. N. Shultze, Acheson.

*Kentucky State Dental Association* Meets annually first Tuesday in June, next meeting at Paris. *Pres.* A. O. Rawls, Lexington; *Sec.* A. W. Smith, Riehmond.

*Lake Erie Dental Association* Meets Annually. *Pres.* C. B. Ansart, Oil City, Pa. *Sec.* C. D. Elliott, Franklin, Pa. Next meeting first Tuesday of May, 1876.

*Mad River Dental Association* Meets at Xenia on the first Tuesdays in April and October. *Pres.* R. Corson, Middletown; *Sec.* E. Betty, Xenia.

*Maine Dental Society*—Meets in Aug., at Thomaston. *Pres.* E. J. Roberts, Augusta; *Sec.* C. E. Hussey, Biddeford.

*Maryland Association of Dentists* Meets the last Thursday of every month. *Pres.* J. B. Bean; *Sec.* S. M. Field.

*Massachusetts Dental Society*—Meets monthly. *Pres.* John T. Codman, Boston; *Rec. Sec.* G. F. Grant, Boston; *Cor. Sec.* T. N, Chandler, Boston.

*Michigan State Dental Association*—Meets annually, next meeting in Detroit in Oct. *Pres.* R. G. Thomas, Detroit; *Rec. & Cor. Sec.* Wm. F. Tremper, Ypsilanti.

*Mississippi Valley Dental Association* Meets annually at Cincinnati, on first Wednesday in March, 1876. *Pres.* H. J. McKellops, St. Louis, Mo.; *Rec. Sec.* Wm. Taft, Cin.

*Mississippi State Dental Association* will meet 3d Wednesday of Aug. '77. *Pres.* J. B. Askew; *Sec.* A. Riser.

*Minnesota Dental Association* Meets annually. Next meeting at Minneapolis; May, 1876. *Pres.* F. A. Williamson, Reading, *Sec.* W. F. Lewis, Winona-St. Paul.

*Missouri Dental Association* Meets on the first Tuesday of June, in St. Louis. *Pres.* C. W. Rivers, St. Louis; *Sec.* W. H. Evans, St. Louis.

*Missouri Valley Dental Association* Meets on the fourth Tuesday in July, 1876, in Omaha, Neb. *Pres.* J. W. Chadduck; *Sec. and Treas.* F. M. Shrirer, Tabor, Iowa.

*Merrimac Valley Dental Association* Meets on the last Thursday of May, 1877, at Portland. *Pres.* A. M. Dudley, Salem, Mass; *Rec. Sec.* W. E. Riggs, Lawrence, Mass., *Cor. Sec.* J. H. Kidder, Lawrence, Mass.

*Memphis Dental Society*--Meets on the first Tuesday of each month. *Pres.* W. M. Arrington; *Sec.* V. F. Elliott; *Treas.* C. L. Harris.

*Nashville Dental Association*—Meets on the second Tuesday of each month. *Pres.* J. C. Ross; *Sec.* R. R. Freeman; *Cor. Sec.* S. J. Cobb.

*New Jersey State Dental Society*—Meets annually. Next meeting at Atlantic City, on the second Tuesday in July, 1876. *Pres.* C. S. Stockton, Newark; *Sec.* C. A. Meeker, Newark.

*New Haven Dental Society*--Meets on the first Wednesday of each month at New Haven. *Pres.* J. T. Metcalf, *Rec and Cor. Sec.* C. L. Smith, of New Haven.

*New Orleans Dental Society* Meets monthly. *Pres.* ———; *Rec. Sec.* A. F. McLane.

*Northern Ohio Dental Association.* Meets annually, second Tuesday of June, 1876, at Cleveland. *Pres.* E. J. Way, Sandusky; *Cor. Sec.* C. Buffett, Clinton.

*Northern Iowa Dental Association*—Meets annually, on the second Tuesday in June, 1873. Next meeting at Waterloo; *Pres.* J. N. Abbott, Lancaster, *Rec. Sec.* A. V Eaton, Anamosa; *Cor. Sec.* A. R. Mason, Waterloo.

*North Carolina Dental Association* Meets annually. Next meeting at Greensboro, 2d Tuesday of Aug., 1876. *Pres.* B. F. Arrington; *Sec.* E. L. Hunter.

*Ohio College Dental Association*--Meets annually Feb. 25. at Cincinnati. *Pres.* N. G. McKellops, St. Louis, Mo.; *Rec. Sec.* H. A. Smith, of Cincinnati.

*Ohio State Dental Association*--Meets annually at Columbus. Next meeting, first Wednesday of December, 1876. *Pres.* C. R. Taft, Cincinnati; *Rec. Sec.* H. L Ambler, Cleveland, O.; *Cor. Sec.* A. F. Emminger, Columbus, O.

*Odontographic Society of Pennsylvania*--Meets on the first Wednesday of each month at 8 o'clock, P. M. in the Philadelphia Dental College. *Pres.* F. M. Dixon; *Cor. Sec.* J. H. McQuillen; *Rec. Sec.* E. L. Hewitt.

*Odontological Society of New York City* Meets the second Tuesday of each month. *Pres.* A. L. Northrup; *Rec. Sec.* Wm. Jarvie, Jr.

*Old Colony Dental Association* Meets quarterly.* *Pres.* C. G. Davis; *Rec. Sec.* L. W. Puffer, North Bridgewater, Mass; *Cor. Sec.* N. C. Fowler, Yarmouth, Mass.

*Ohio Valley Dental Association* Meets annually, in N. E. Ky; this year in Paris, Ky. Tuesday, Sept. 9th.

*Oregon State Dental Society* Meets annually. *Pres.* J. R. Cardwell, Portland; *Rec. Sec.* G. N. Chance, Portland.

*Pennsylvania State Dental Society* meets next in Minnequa Sprins, 2d Tuesday o July, 1877. *Pres.* J. S. King, Pittsburg; *Rec. Sec.* N. S. Guilford, Philadelphia; *Cor Sec.* M. H. Webb, Lancaster.

*Pennsylvania Association of Dental Surgery* Meets monthly in Philadelphia. *Pres* Spencer Roberts; *Sec.* G. Pettit.

PITTSBURG DENTAL SOCIETY—Meets second Tuesday of each month. *Pres.* W. F Fundenburg; *Sec.* M. L. King.

*San Francisco Dental Association* Meets monthly. *Pres.* C. C. Knowles; *Cor. Se* H. E. Knox; *Rec. Sec.* Wm. Dutch.

*St. Louis Dental Society* Meets monthly. *Pres.* W. H. Morrison; *Sec.* A. H. Fuller.

*Susquehanna Dental Association* Meets annually. *Pres.* W. H. Wissimer, Williamsport, Pa.; *Rec. Sec.* W H. Hertz. Meets May, 1876, at Pittston.

*Society of Dental Surgeons of the City of New York* Meets semi-monthly in New York. *Pres.* B. F. Franklin: *Rec. Sec.* J. S. Latimer; *Cor. Sec.* E. H. Burras.

**Southern Dental Association* Meets annually. Next meeting at Nashville, Tenn, at time of Mardi-Gras. *Pres.* W. G. Redman, La.; *Rec. Sec.* J. F Thompson. Va

*South Carolina State Dental Association* Meets annually. *Pres.* G. W. Norwood, *Sec.* G. F. S. Wright, *Cor. Sec.* N. D. Wilson. Next meeting at Columbia, the first Tuesday of June, 1877.

*Tennessee Dental Association* Meets semi-annually. Next meeting in Nashville, on the 4th Wednesday in June. 1876. *Pres.* R. R. Freeman; *Rec. Sec.* W. H. Morgan *Cor. Sec.* L. G. Noel.

*Virginia State Dental Association* Meets in Richmond, first Thursday in Nov. o each year. *Pres.* H. M. Grant, Abington: *Sec.* G. F. Keesee, Richmond.

*Washington, City Dental Society* Meets annually. *Pres.*, R. F. Hunt, *Sec.* Dr. Evans

*Wisconsin State Dental Association* Meets annually. Next meeting at Milwaukee second Tuesday of July, 1876. *Pres*, R. S. Wells, Sparta; M. T. Moore, La Crosse.

### State and District Dental Societies of State of New York.

*New York State Dental Society*--Meets at Albany on the second Wednesday of July next. *Pres.* W. C. Barrett, Warsaw, N. Y.; *Cor. Sec.* S. B. Palmer; *Rec. Sec.* S. S. A, Freeman, Buffalo.

*First District* Meets second and fourth Wednesday evenings of each month. Annual meeting in New York, first Tuesday in June. *Pres.* J. S. Latimer New York, *Rec. Sec.* F. M. Odell, New York.

*Second District.* Annual meeting in Brooklyn, first Monday in April. *Pres.* W. S. Elliott; *Rac. Sec*, M. E. Elmandorf. Brooklyn; *Cor. Sec.* F. W. Dalbare.

*Third Distriet.* Meets semi-annually on second Tuesday in January and July. Annual meeting at Albany on second Tuesday in January. *Pres.* S. P. Welsh; *Rec. Sec.* H. A. Hall, Troy; *Cor. Sec.* J. A. Perkins, Albany.

*Fourth District.* Meets semi-annually. Meets annually at Saratoga on second Tnesday in June. *Pres.* C, A. Elmore, Ft. Edward; *Rec. Sec.* E. S. Pearsall, Saratoga.

*Fifth District.* Meets semi-annually. Annual meeting at Syracuse. second Tuesday in June. *Pres.* F. D. Nellis, Syracuse; *Rec. Sec.* J. S. Marshall, Syracuse; *Cor. Sec.* A. B. Cowles, Rome.

*Sixth District.* Meets semi-annually. Annual meeting at Binghampton on second Tuesday in June, *Pres.* Frank B. Darby, Oswego; *Rec. Sec.* F. E. Perry, Norwich *Cor. Sec.* A. M. Holmes, Morrisville.

*Seventh District*, Meets semi-annually. Annual meeting at Rochester first Tuesday In June. *Pres.* H. S. Miller; *Rec. Sec.* J. E. Line, Rochester.

*Eighth District.* Meets semi-annually. Annual on second Tuesday in May. *Pres.* G. C. Daboll, *Rec. Sec.* S. A. Freeman, *Cor. Sec.* T. G. Lewis, Buffalo.

*The Seventh and Eighth District Societies* hold a union meeting on the last Tueday of October, alternately at Buffalo and Rochester.

The Ohio State Board of Dental Examiners will meet in Cincinnati on the first Wednesday of March 1876, at 12 o'clock. *Pres.* J. Taft; *Sec.* W. P. Horton.

### Exchanges.

ST. LOUIS MEDICAL AND SURGICAL JOURNAL.
BOSTON MEDICAL AND SURGICAL JOURNAL.
THE PACIFIC MEDICAL AND SURGICAL JOURNAL, **SAN FRANCISCO.**
PHILADELPHIA MEDICAL AND SURGICAL JOURNAL.
CINCINNATI MEDICAL ADVANCE.
CINCINNATI LANCET AND OBSERVER.
DENTAL COSMOS, PHILADELPHIA,
LADIES' REPOSITORY, CINCINNATI.
HALL'S JOURNAL OF HEALTH, N. Y.
CHICAGO MEDICAL EXAMINER,
THE DETROIT REVIEW OF MEDICINE **AND PHARMACY.**
MEDICAL RECORD, N. Y.
NASHVILLE JOURNAL OF MEDICINE AND SURGERY.
AMERICAN JOURNAL OF DENTAL SCIENCE, BALTIMORE.
THE UNITED STATES MEDICAL AND SURGICAL JOURNAL, **CHICAGO.**
NEW ORLEANS JOURNAL OF MEDICINE,
AMERICAN AGRICULTURIST, N. Y.
BOSTON JOURNAL OF CHEMISTRY.
JOURNAL OF APPLIED CHEMISTRY, N. Y.
CANADA JOURNAL OF DENTAL SCIENCE, **MONTREAL.**
INDIANA JOURNAL OF MEDICINE,
MEDICAL AND SURGICAL REPORTER. **SALEM, OREGON.**
MISSOURI DENTAL JOURNAL, ST. LOUIS.
ECLECTIC MEDICAL JOURNAL, CINCINNATI.

### HARVARD UNIVERSITY.

### Dental Department.

### BOSTON, MASS. 1875–76.

---

### FACULTY.

CHARLES WILLIAM ELIOT, L.L.D., *President.*
OLIVER W. HOLMES, M.D., Professor of Anatomy.
HENRY J. BIGELOW, M.D., Professor of Surgery and Clinical Surgery.
THOMAS H. CHANDLER, D.M.D., Professor of Mechanical Dentistry.
GEORGE T. MOFFATT, M.D., D.M.D., Professor of Operative Dentistry.
LUTHER D. SHEPARD, D.D.S., Adjunct Professor of Operative Dentistry.
NATHANIEL W. HAWES, Assistant Professor of Operative Dentistry.
EDWARD S. WOOD, M.D., Assistant Professor of Chemistry.
HENRY P. BOWDITCH, M.D., Assistant Professor of Physiology.
WILLIAM H. ROLLINS, D.M.D., Instructor in Dental Pathology.
CHARLES A. BRACKETT, D.M.D., Instructor in Dental Therapeutics.
IRA A. SALMON, D.D.S., University Lecturer on Operative Dentistry.
CHARLES B. PORTER, M.D., Demonstrator of Practical Anatomy.
CHARLES WILSON, D.M.D., Demonstrator in Charge.
GEORGE F. GRANT, D.M.D., Demonstrator of Mechanical Dentistry.

---

The Ninth year of this School begins on the 28th of September and continues till the last Wednesday in June. The distinction of Summer and Winter sessions being hereafter abolished. It will be divided into two equal terms, with a recess of one week at Christmas. The course of instruction will be progressive, extending over two years, the lectures of one year not being repeated in the next.

The regular examinations will be held in the following order, viz.: At the end of the first year, anatomy, including dissection, physiology and general chemistry. A certificate from the demonstrator of anatomy will be required of each student, that he has satisfactorily dissected the three parts of the body.

At the end of the second year, dental pathology, including a knowledge of gestation and diseases of women so far as they affect the mouth and throat, dental materia medica and therapeutics, oral surgery and surgical pathology, operative and mechanical dentistry. The examinations in operative and mechanical dentistry will include actual operations and the preparation of specimens of mechanical dentistry.

Every candidate must be twenty-one years of age, and of good moral character; he must give evidence of having studied medicine or dentistry three full years; he must have spent at least one continuous year at this school, have presented a satisfactory thesis and have passed all the required examinations.

The University Degree, D.M.D. (*Dentariæ Medicinæ Doctor*), is conferred upon those who fulfil the requirements.

Students may be admitted to advanced standing upon passing a satisfactory examination in a majority of the studies already pursued by the class; but no student shall advance with his class or be admitted to advance standing until he has passed such examination.

The work in the operative and mechanical infirmaries will go on throughout the course, but no student shall be permitted to operate at the chair until he has by observation and practice on extracted teeth satisfied the Professor of his fitness.

The faculty recommend young men who propose to take the degree to spend the whole of the required term of three years of study in the school. But those who wish to spend but two of the three years in the school are earnestly advised to pass their first year of study, before entering, under the direction of a *competent* private instructor.

Unsurpassed facilities for operative and mechanical practice are furnished by the infirmary of the Massachusetts General Hospital which is open throughout the year.

Jan.

---

### FEES.

There are no fees for matriculation, for the diploma, nor for the demonstrators. For the first year a student is a member of the school, the fee is $200; for the second year, $150; for any subsequent year, $50.

For further information address,

**THOS. H. CHANDLER, Dean,**

**222 Tremont Street, Boston, Mass.**

